# Machine Learning-Based Multidimensional Health Decline Prediction Framework: Data-Driven Modeling for the Middle-Aged and Elderly Population

**DOI:** 10.3390/healthcare14142223

**Published:** 2026-07-22

**Authors:** Xiaomin Li, Xudong Guo, Xufeng Fu

**Affiliations:** 1 Key Laboratory of Fertility Preservation and Maintenance of Ministry of Education, Ningxia Medical University, Yinchuan 750004, China; lixm@nxmu.edu.cn; 2 College of Information Engineering, Northwest A&F University, Yangling 712100, China

**Keywords:** CHARLS database, multidimensional health outcomes prediction, machine learning, elderly health, Shapley

## Abstract

**Highlights:**

**What are the main findings?**

- A comprehensive evaluation of 15 machine learning algorithms was conducted, with TabPFN demonstrating the best performance in effectively predicting health conditions such as disability, pain, cognitive impairment, hearing, and depression.
- SHAP analysis identified key predictive factors including Sleep quality, Daytime napping, Hearing, Marital satisfaction, Smoking status, Marital status, Activities of Daily Living (ADL), Walking speed and HbA1c, revealing common risk characteristics associated with multiple health outcomes.

**What are the implications of the main findings?**

- The TabPFN model provides a reliable tool for early screening and accurate prediction of various health risks in middle-aged and elderly individuals.
- This study identified key modifiable factors that provide a scientific basis for individualized health management, targeted interventions, and the development of healthy aging strategies.

**Abstract:**

**Background:** With the worsening of global population aging, individuals aged 45 and above face numerous challenges, including disability, pain, cognitive impairment, hearing, and depression. This study aims to utilize machine learning and deep learning methods to develop predictive models for forecasting the health outcomes and disease risk among middle-aged and elderly individuals, while identifying the key factors influencing disease prevalence and quality of life in this demographic. **Methods:** This research is grounded in the China Health and Retirement Longitudinal Study (CHARLS) database (2015–2018), encompassing 20,967 participants. Machine learning and deep learning techniques were employed to create predictive models for disability, pain, cognitive impairment, hearing, and depression, utilizing 69 variables. A minimum redundancy maximum relevance (MRMR) feature selection strategy and incremental feature selection (IFS) were applied to identify key variables. The optimal predictive models were evaluated for accuracy using the area under the receiver operating characteristic curve (ROC-AUC). **Results:** The analysis resulted in 15 predictive models, with TabPFN showing the highest performance in predicting disability, pain, cognitive impairment, hearing, and depression, achieving AUCs of 0.802, 0.860, 0.814, 0.769, and 0.856, respectively. Shapley interpretability analysis identified the top five critical features affecting these health outcomes. **Conclusions:** The proposed models can assess the disease risk of various conditions in middle-aged and elderly individuals, suggesting that early prevention and effective intervention may reduce incidence rates. Additionally, the study provides insights into key factors influencing health, offering a scientific foundation for tailored health management and precision intervention strategies.

## 1. Introduction

According to data from the World Health Organization, one-sixth of the global population is projected to be aged 60 years or older by 2030, and the number of people aged 60 years and above worldwide will double to 2.1 billion by 2050, reflecting a rapidly aging global population [1]. In the United States, 41.7% of adults aged 65 years and older are identified as having a disability [1]. As of January 2020, the number of disabled elderly individuals aged 60 and above in China has surpassed 42 million, and it is projected to increase to 77 million by 2030 [2]. Among the diseases affecting elderly individuals reported in various studies, the incidence of disability is notably high, leading to significantly diminished independence and quality of life, as well as an accompanying rise in mortality rates [3]. Additionally, chronic pain affects over 50% of the older population and up to 80% of nursing home residents impacted [4]. European survey-based studies indicate that the prevalence of chronic pain increases with age, with an estimated incidence rate of 38% to 60% among those aged 65 and older [4]. Meanwhile, millions of people worldwide suffer from cognitive impairments and dementia, along with their caregivers, who endure considerable distress [5]. Furthermore, hearing loss in the elderly has emerged as a serious public health issue today. Research predicts that the number of cases of hearing loss among older adults globally will gradually rise from 2020 to 2040. Before 2020, the average annual growth rate of global hearing loss cases was 2.42%, and the age standardized rate (ASR) increased by 0.08% annually [6]. Population aging is identified as a primary driving factor for hearing loss, accounting for 60.83% and 35.35% of the respective rates [6]. Depression is a common emotional disorder among the elderly and poses a widespread public health challenge. Studies focusing on individuals aged 60 and above indicate that the overall prevalence of clinically diagnosed depressive disorders is approximately 5.7%, with the incidence of depression increasing with age, peaking in the population aged 85 and older [7]. These diseases in the elderly not only inflict physical and psychological suffering on patients but also impose significant caregiving and economic burdens on families and society, straining healthcare systems. Therefore, accurately identifying high-risk conditions related to disabilities, pain, cognitive impairment, hearing and depression in the elderly, and devising personalized treatment and preventive strategies at an early stage, is crucial for improving the quality of life and reducing mortality rates among older adults.

Previous research on predicting disabilities, pain, and depression among the elderly primarily utilized traditional logistic regression statistical methods. While this approach performs well in handling binary classification problems, it frequently overlooks the temporal dependencies and higher-order interactions of risk factors, areas in which machine learning models demonstrate proficiency [8,9]. Moreover, machine learning demonstrates excellent scalability, continuously optimizing performance and adapting to new tasks as data volume increases. These advantages have led to machine learning outperforming traditional statistical methods in many practical applications, particularly within big data environments.

Although some studies have employed machine learning algorithms to establish predictive models for disability and cognitive impairment in the elderly, there remain deficiencies in identification performance and predictive accuracy. Previous studies have used eXtreme Gradient Boosting(XGBoost) algorithm to establish a disability prediction model for the elderly (AUC of 0.724) and CNN BiLSTM Attention algorithm to establish a cognitive impairment risk prediction model for the elderly (AUC of 0.73), both of which show low accuracy [9,10]. There are also studies using Gradient Boosting Machine to establish hearing prediction models (training AUC 0.887 vs. testing AUC 0.678) [11], but the models have overfitting issues. Therefore, developing accurate predictive models for assessing disability, pain, cognitive impairment, hearing, and depression in the elderly population is of great significance for prevention and care of the elderly.

The present study utilized the CHARLS database to obtain a sample dataset of 20,967 observations to construct a predictive model for the health outcomes of elderly individuals. The dataset comprises 69 attributes, including demographic characteristics, physical examination results, medication usage, chronic diseases, laboratory results, satisfaction levels, and lifestyle factors. This study introduced Tabular Prior-Data Fitted Network (TabPFN), a lightweight and rapid prediction algorithm, combined with Shapley values, to create more interpretable predictive models for five distinct health outcomes. TabPFN is an innovative deep learning model specifically designed for tabular data. It has been trained on millions of synthetic datasets with the aim of enhancing the ability to decipher complex data patterns [12]. Compared with conventional machine learning algorithms, TabPFN is based on a pretrained Transformer architecture and performs prediction directly through Bayesian inference without requiring a complex model optimization process. This enables efficient learning on medium-sized structured tabular datasets while achieving strong predictive performance. In clinical research, previous studies have reported that TabPFN achieved an AUC of 0.953 for the early prediction of acute kidney injury [13]. PyCaret is an open-source, low-code machine learning library that automates the machine learning workflow and employs cross-validation methods for comprehensive evaluation of various machine learning algorithms [14]. In addition, PyCaret includes approximately 75 machine learning algorithms. In this study, representative algorithms were selected, covering linear models, probabilistic classifiers, distance-based methods, decision trees, ensemble learning methods, and support vector machines. This model selection strategy encompasses diverse machine learning paradigms, enabling a comprehensive comparison across different algorithms and providing a fair and consistent benchmark for evaluating the performance of TabPFN. Overall, fourteen machine learning algorithms were comprehensively evaluated in this study, which included Logistic Regression (LR), K-Nearest Neighbors Classifier (KNN), Naive Bayes, Decision Tree Classifier, Support Vector Machine with Linear Kernel (SVM—Linear), Support Vector Machine with Radial Basis Function (SVM—RBF), Ridge Classifier, Random Forest Classifier, Linear Discriminant Analysis (LDA), Quadratic Discriminant Analysis (QDA), AdaBoost Classifier, Gradient Boosting Classifier (GBM), Extra Trees Classifier (Extremely Randomized Trees), CatBoost Classifier, LightGBM, and Dummy Classifier. Shapley Additive exPlanations (SHAP) is one of the most popular feature-based machine learning interpretability methods. It can be seamlessly integrated into supervised machine learning models to gain deeper insights into their predictions, thereby enhancing their transparency and trustworthiness [15]. Based on the above methods, we aim to provide clinicians and public health workers with an intuitive risk assessment tool, enabling them to quickly and effortlessly evaluate five health risks of disability, pain, cognitive impairment, hearing, and depression in the elderly. This tool will also enable the precise identification of high-risk populations, thereby offering robust support for their treatment and prevention strategies.

## 2. Materials and Methods

### 2.1. Data Sources

The data used in this study were obtained from the China Health and Retirement Longitudinal Study (CHARLS), which is publicly available at http://charls.pku.edu.cn. CHARLS, conducted by the National School of Development at Peking University, is a nationally representative longitudinal survey of the Chinese population aged 45 years and older. It was established to develop a high-quality public microdatabase that collects information on multiple domains, including socioeconomic status and health outcomes, to support aging-related research [16]. The study population consists of a total of 20,967 middle-aged and older adults in China, among which 2917 are disabled, 5843 report pain in a specific part of their body, 1229 face cognitive impairments, 7259 have hearing impairments, and 2169 are exhibiting signs of depression.

### 2.2. Definition of Outcome Variables

Disability: Disability is defined as the presence of any one of the following conditions—physical disability, brain injury/intellectual disability, blindness or partial blindness, deafness or partial deafness, mutism, or severe stuttering—provided that the individual was not disabled in the last survey but is reported as disabled in the current survey. In this study, disability was coded as “1” and no disability as “0”.

Pain: Any reported pain in a specific part of the body is classified as pain. In this study, pain was coded as “1” and no pain as “0”.

Cognitive Impairment: Cognitive impairment is assessed based on evaluations of time orientation, calculation ability, and memory. Time orientation involves responding correctly to questions about the year, month, day, season, and day of the week, earning 1 point for each accurate response, with a total score ranging from 0 to 5. Calculation ability is assessed by subtracting 7 from 100, continuing to subtract 7 for a total of seven times, with 1 point awarded for each correct answer, resulting in a score from 0 to 5. Participants are also asked to reproduce a provided image, scoring a total of 0 or 1. Memory is evaluated through the recall of a list of words, scoring between 0 and 12. Lastly, a number pattern completion task scores from 0 to 3. The total possible score is 26 points; a score of ≥18 indicates no cognitive impairment, while a score of <18 indicates a risk for cognitive impairment. In this study, cognitive impairment was coded as “1” and no cognitive impairment as “0”.

Hearing: Hearing ability in this study is classified as either good or poor. Individuals who do not wear hearing aids and report their hearing as excellent, very good, good, or fair are classified as “good”, while those not wearing hearing aids who report their hearing as poor are classified as “poor”. In this study, hearing impairment was coded as “1” and no hearing impairment as “0”.

Depression: Symptoms of depression are assessed using the Center for Epidemiologic Studies Depression Scale (CES-D), which consists of 10 items. Responses are scored as follows: rarely or none of the time (<1 day) is 0 points, a little of the time (1–2 days) is 1 point, some or half of the time (3–4 days) is 2 points, and most of the time (5–7 days) is 3 points. The total score ranges from 0 to 15 points, indicating no or minimal symptoms of depression. Scores between 16 and 30 indicate mild to moderate to severe depression. In this study, we modeled depression as a binary category, with a label value of 1 indicating depression and 0 indicating no depression.

### 2.3. Definition of Covariates

Covariates were categorized into demographic characteristics, lifestyle and health behaviors, physical examination indicators, activities of daily living, medication use, chronic diseases, laboratory biomarkers, and satisfaction-related variables.

#### 2.3.1. Demographic Characteristics

Demographic variables included age, gender, body mass index (BMI), and marital status. Age was restricted to participants aged 45 years or older. Gender was classified as male or female. BMI was calculated as weight (kg) divided by height squared (m^2^). Marital status was categorized as married, cohabiting, currently not living with a spouse, separated, divorced, widowed, or unmarried.

#### 2.3.2. Lifestyle and Health Behaviors

Lifestyle-related variables included smoking status, alcohol consumption, sleep quality, daytime napping, and social participation.

Smoking status was classified as having a smoking history (current or former smoker) or no smoking history (never smoker). Alcohol consumption was categorized as current/former drinkers or never drinkers.

Sleep quality was assessed using the question: “During the past month, how many hours of actual sleep did you get at night on average?” Daytime napping was determined by the question: “During the past month, how long did you usually nap after lunch?”

Social participation was evaluated based on engagement in social activities, with scores assigned according to the number of activities in which participants participated.

#### 2.3.3. Physical Examination Indicators

Physical examination variables included systolic blood pressure (SBP), diastolic blood pressure (DBP), pulse rate, respiratory function, left- and right-hand grip strength, standing balance, walking speed, upper arm length, knee height, waist circumference, complete tooth loss, history of cataract surgery, glaucoma, myopia, hyperopia, hearing, and use of assistive devices.

SBP, DBP, pulse rate, and respiratory function were measured three times, and the average value was used for analysis.

Grip strength was calculated as the mean of two measurements for each hand.

Standing balance was defined as the ability to maintain any of the following positions for 10 s: semi-tandem stand, tandem stand, or side-by-side stand.

Walking speed was calculated as the average time from two usual-pace walking tests.

Upper arm length and knee height were measured using a Martin anthropometer.

Complete tooth loss, cataract surgery history, and glaucoma were coded as binary variables (yes = 1, no = 0).

Visual function was assessed using self-reported distance vision (myopia) and near vision (hyperopia). Responses of “excellent”, “very good”, or “good” were categorized as good vision, whereas “fair” or “poor” were categorized as poor vision.

Hearing was assessed using self-reported hearing ability. Participants reporting poor hearing or requiring hearing assistance were classified as having hearing impairment.

Use of assistive devices was defined as the use of any mobility or daily-living aid, including a cane, walker, manual wheelchair, electric wheelchair, catheter, urine collection bag, or portable toilet.

#### 2.3.4. Activities of Daily Living

Activities of daily living (ADL) were assessed using the Katz ADL Scale, with total scores ranging from 0 to 6. Higher scores indicated greater functional independence.

#### 2.3.5. Medication Use

Medication-related variables included the use of prostate medications (2015), antihypertensive medications (2018), antidiabetic medications (2018), anticancer medications (2018), and analgesics (2018). All medication variables were coded as binary variables (yes = 1, no = 0).

#### 2.3.6. Chronic Diseases

Chronic conditions included hypertension, dyslipidemia, diabetes mellitus, malignant tumors, chronic lung disease, liver disease, heart disease, stroke, kidney disease, gastrointestinal disease, emotional or psychiatric disorders, memory-related disorders, arthritis, and asthma.

All chronic diseases were based on self-reported physician diagnoses and were coded as binary variables (yes = 1, no = 0).

#### 2.3.7. Laboratory Biomarkers

Laboratory biomarkers included white blood cell count (WBC), hemoglobin (Hb), hematocrit (Hct), mean corpuscular volume (MCV), platelet count (PLT), triglycerides (TG), creatinine (Cr), high-density lipoprotein cholesterol (HDL-C), low-density lipoprotein cholesterol (LDL-C), total cholesterol (TC), glucose (GLU), uric acid (UA), cystatin C (Cys C), C-reactive protein (CRP), and glycated hemoglobin (HbA1c).

#### 2.3.8. Satisfaction-Related Variables

Satisfaction-related variables included satisfaction with health, marriage, and overall life. Responses of “excellent”, “very good”, or “good” were categorized as satisfactory, whereas responses of “fair” or “poor” were categorized as unsatisfactory.

### 2.4. Data Preprocessing

**Figure 1:**
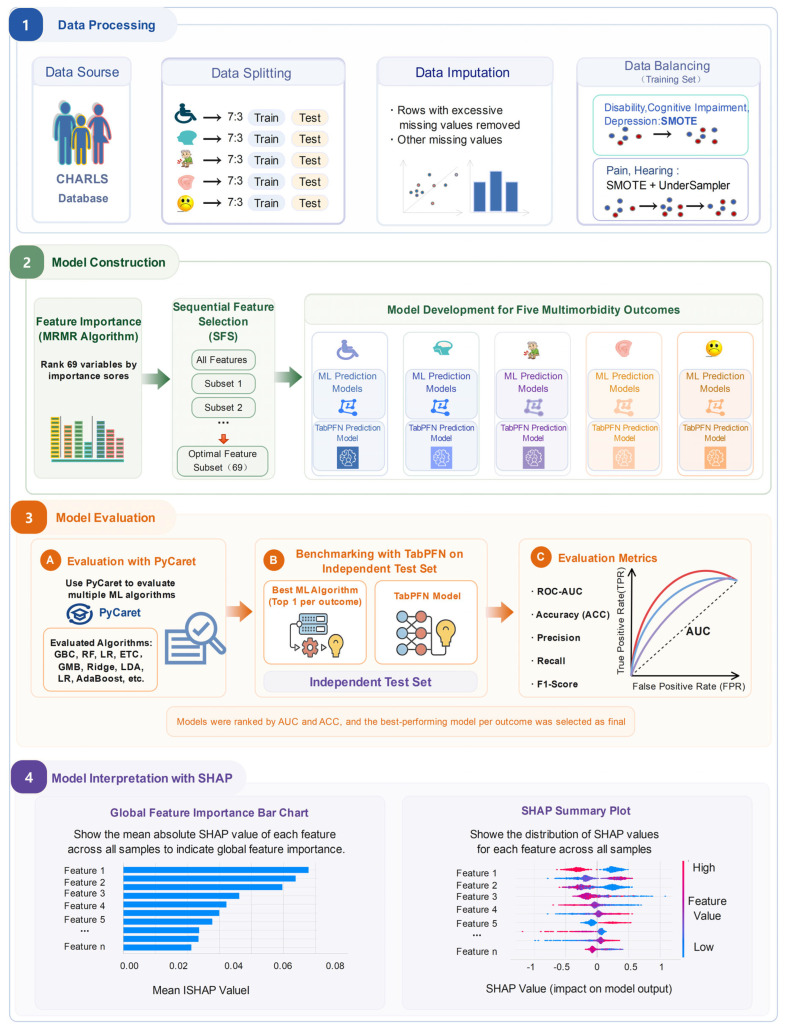
Research Workflow. (1) Data acquisition from CHARLS; (2) Model development using TabPFN and PyCaret; (3) Model Evaluation; (4) SHAP explanation.

Following the workflow of this study (shown in Figure 1), to meet the requirements of model training, the dataset was first randomly divided into training and testing sets using a stratified random sampling strategy at a ratio of 7:3. Subsequently, missing variables in both the training and testing sets were preprocessed. Some variables in the dataset contained missing values, and directly removing samples with missing data could introduce selection bias and affect the generalizability and accuracy of the study findings. Therefore, for variables with missing values (e.g., walking speed, arm length, and waist circumference), regression models were established using the training set to predict missing values, and the trained models were then applied to the testing set for imputation. For variables with a relatively low proportion of missing values (e.g., laboratory measurements), mean imputation based on the training set was applied.

Considering the imbalance between positive and negative samples across different prediction tasks, the disability dataset contained 2917 positive samples, the pain dataset contained 5843 positive samples, the cognitive impairment dataset contained 1229 positive samples, the hearing impairment dataset contained 7259 positive samples, and the depression dataset contained 2169 positive samples. In this study, class balancing was performed only on the training sets. For the prediction tasks of disability, cognitive impairment, and depression, the Synthetic Minority Over-sampling Technique (SMOTE) was applied to moderately oversample positive samples in the training sets, adjusting the ratio of positive to negative samples to approximately 1:2. For the hearing impairment and pain prediction tasks, random undersampling (UnderSampler) was further combined with SMOTE-based oversampling to moderately reduce negative samples, resulting in a final training set class ratio of 1:1. The independent testing sets remained completely separate throughout the data processing, model training, and model selection procedures and were used only for final model performance evaluation, without any oversampling, undersampling, or data augmentation operations.

### 2.5. Model Construction

For the balanced training sets of the five health conditions (disability, pain, cognitive impairment, hearing impairment, and depression), the minimum redundancy maximum relevance (MRMR) algorithm was first applied to rank the importance of 69 candidate features. Subsequently, the incremental feature selection (IFS) method was employed to progressively increase the number of selected features. By comparing the average model performance of different feature subsets through 10-fold cross-validation, the optimal number of features for each prediction model was determined. During the construction of the candidate feature sets, corresponding questionnaire items and their derived variables related to each outcome were excluded from the 69 candidate variables to prevent label leakage. Specifically, the outcome variables for each health condition were independently defined based on CHARLS questionnaire items, including disability-related items, the CES-D scale, cognitive function assessment items, hearing status items, and pain-related items. These outcome-defining items and their derived variables were not included in the MRMR feature selection process. For each prediction task, one health outcome was designated as the prediction target, and the remaining 68 variables were used as input features for model training.

After determining the optimal feature subset for each health condition, TabPFN and traditional machine learning models were applied for model construction. TabPFN was directly implemented using the official pretrained weights, with model parameters kept fixed during inference. Traditional machine learning models were optimized and evaluated using the PyCaret platform. The hyperparameter optimization process was performed exclusively on the training sets through cross-validation.

Finally, all candidate models were evaluated on the independent testing sets. The optimal model for each health condition prediction task was selected based on a comprehensive ranking of the area under the receiver operating characteristic curve (AUC) and accuracy (ACC). In addition, the SHapley Additive exPlanations (SHAP) method was applied to interpret the final selected models.

To prevent data leakage, all feature selection procedures (MRMR and IFS), hyperparameter optimization, and data balancing operations were performed exclusively on the training sets. The independent testing sets remained completely isolated until the final model evaluation and were not involved in any model training or parameter tuning procedures.

All analyses were performed using Python. The primary machine learning models were implemented using PyCaret and scikit-learn. TabPFN was implemented using the official pretrained model provided by the developers. Model interpretability was performed using the SHAP library. All experiments were conducted on a server equipped with an Intel Core i7-13700K CPU @ 3.40 GHz, 32 GB RAM, and an NVIDIA GeForce RTX 4060 Ti GPU.

### 2.6. Model Evaluation

For each prediction task, the PyCaret low-code platform was first used to comprehensively evaluate 14 machine learning algorithms using 10-fold cross-validation, including Ridge, Gradient Boosting Classifier (GBC), Light Gradient Boosting Machine (LightGBM), Linear Discriminant Analysis (LDA), Logistic Regression (LR), Random Forest (RF), AdaBoost, Extra Trees Classifier (ETC), Naïve Bayes (NB), Decision Tree (DT), Support Vector Machine with a linear kernel (Linear SVM), K-Nearest Neighbors (KNN), Quadratic Discriminant Analysis (QDA), and Dummy Classifier. The best-performing machine learning algorithm was then selected for each health outcome.

Subsequently, the optimal ML algorithms and TabPFN were evaluated on an independent test set. The rankings were established based on the evaluation metrics AUC and ACC, leading to the construction of optimal predictive models for each target. The evaluation metrics employed in this study included the area under the ROC curve (AUC), accuracy, recall, and F1-Score. The formulas are as follows:

(1) $$\mathrm{Accuracy}=\frac{\mathrm{TP}+\mathrm{TN}}{\mathrm{TP}+\mathrm{TN}+\mathrm{FP}+\mathrm{FN}}$$

(2) $$\mathrm{Sensitivity}=\frac{\mathrm{TP}}{\mathrm{TP}+\mathrm{FN}}$$

(3) $$\mathrm{Precision}=\frac{\mathrm{TP}}{\mathrm{TP}+\mathrm{FP}}$$

(4) $$\mathrm{MCC}=\frac{\mathrm{TP}\times \mathrm{TN}-\mathrm{FP}\times \mathrm{FN}}{\sqrt{(\mathrm{TP}+\mathrm{FP})\times (\mathrm{TP}+\mathrm{FN})\times (\mathrm{TN}+\mathrm{FP})\times (\mathrm{TN}+\mathrm{FN})}}$$

(5) $$F1=\frac{2\times \mathrm{Precision}\times \mathrm{Recall}}{\mathrm{Precision}+\mathrm{Recall}}$$

Here, TP, TN, FP, and FN represent the numbers of true positive, true negative, false positive, and false negative samples, respectively, with their full forms being True Positive, True Negative, False Positive, and False Negative.

### 2.7. Statistics

We performed statistical analysis on the collected data. For continuous variables, we described their central tendency and dispersion using mean ± standard deviation (SD) to better understand the distribution of the data. For categorical variables, we presented the distribution of each category using frequency and percentage, indicated as frequency (%). All statistical analyses were conducted using Python (Version 3.12.0; Python Software Foundation, 2023). A *p*-value of less than 0.05 was considered statistically significant.

## 3. Results

### 3.1. Baseline Characteristics and Disease Incidence

Table 1 presents the statistical analysis results of the incidence rates of disabilities and related variables. The disability prevalence rate among older adults in this study was 13.91% (2917/20,967). The characteristics associated with disability included sleep quality, activities of daily living (ADL) capabilities, hearing, analgesics use, chemotherapy drugs, pain severity, cataract surgery, glaucoma, vision issues (myopia/hyperopia), kidney disease, cognitive impairment, hypertension, diabetes, lung disease, heart disease, stroke, emotional disorders, memory-related disorders, use of assistive devices, and tooth loss. These variables showed significant differences between the disabled and non-disabled groups (*p* < 0.001). (Refer to Table 1).

The prevalence of pain was 27.86% (5843/20,967), with associated characteristics including sleep quality, daytime napping, ADL, analgesics use, cognitive impairment, glaucoma, vision, hypertension, diabetes, lung disease, liver disease, heart disease, kidney disease, gastrointestinal disorders, memory-related disorders, emotional disorders, arthritis, asthma, use of assistive devices, and tooth loss. All of these variables exhibited significant differences between the pain and non-pain groups (*p* < 0.001) (Refer to Supplementary Table S1).

Regarding cognitive impairment, the prevalence rate was 5.86% (1229/20,967). Statistical analysis indicated that waist circumference, diastolic blood pressure, left-hand grip strength, MVC, TG, sleep quality, ADL, analgesics use, pain, glaucoma, vision, dyslipidemia, memory-related disorders, asthma, use of assistive devices, and tooth loss. All these variables demonstrated significantly differed between the cognitive impairment and non-cognitive impairment groups (*p* < 0.005) (Refer to Supplementary Table S2).

The prevalence of hearing impairment was 34.62% (7259/20,967), and its associated characteristics included LDL, TC, sleep quality, ADL, life satisfaction, analgesics use, pain, cataract surgery, glaucoma, vision, cognitive impairment, disability, heart disease, kidney disease, stroke, gastrointestinal disorders, memory-related disorders, emotional disorders, asthma, use of assistive devices, and tooth loss. These variables showed significant differences between the hearing impairment and non-hearing impairment groups (*p* < 0.005) (Refer to Supplementary Table S3).

The prevalence of depression was 10.34% (2169/20,967), with associated characteristics including waist circumference, diastolic blood pressure, respiratory status, right-hand grip strength, walking speed, BMI, Hb, Hct, MCV, Cr, HDL, UA, CRP, daytime napping, gender, marital status, alcohol consumption, social participation, health satisfaction, marital satisfaction, life satisfaction, antihypertensive medications, diabetes medications, cataract surgery, analgesics, stroke, and asthma. All these variables demonstrated significant differences between the depression and non-depression groups (*p* < 0.005) (Refer to Supplementary Table S4).

The above statistical analysis results indicate that sleep quality, ADL, analgesics use, Glaucoma, vision, memory-related disorders, use of assistive devices, asthma, pain, and cognitive impairment are likely important factors influencing these five health conditions.

### 3.2. Model Evaluation and Comparison

**Figure 2:**
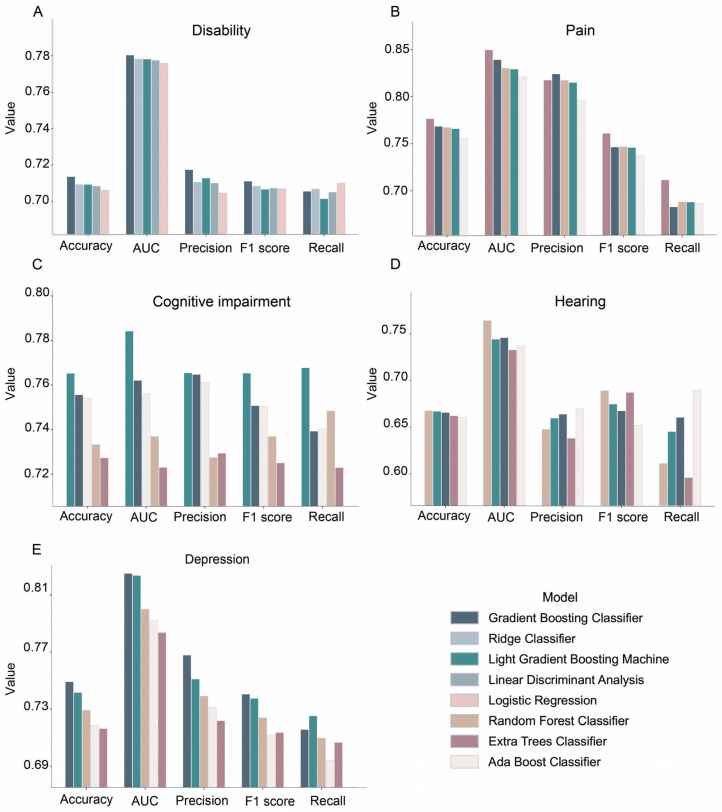
Performance comparison of five machine learning algorithms across five health states on the validation set. (**A**) Disability; (**B**) Pain; (**C**) Cognitive Impairment; (**D**) Hearing; (**E**) Depression.

First, for the prediction of the five health outcomes (disability, pain, cognitive impairment, hearing impairment, and depression), 14 machine learning algorithms, including Ridge, GBC, LightGBM, LDA, LR, RF, AdaBoost, ETC, NB, DT, Linear SVM, KNN, QDA, Dummy, were comprehensively evaluated using 10-fold cross-validation. As shown in Figure 2, the performance of the top five machine learning algorithms for each health outcome is presented. Among them, GBC, ETC, LightGBM, RF, and GBC achieved the best performance for predicting disability, pain, cognitive impairment, hearing impairment, and depression, respectively.

**Figure 3:**
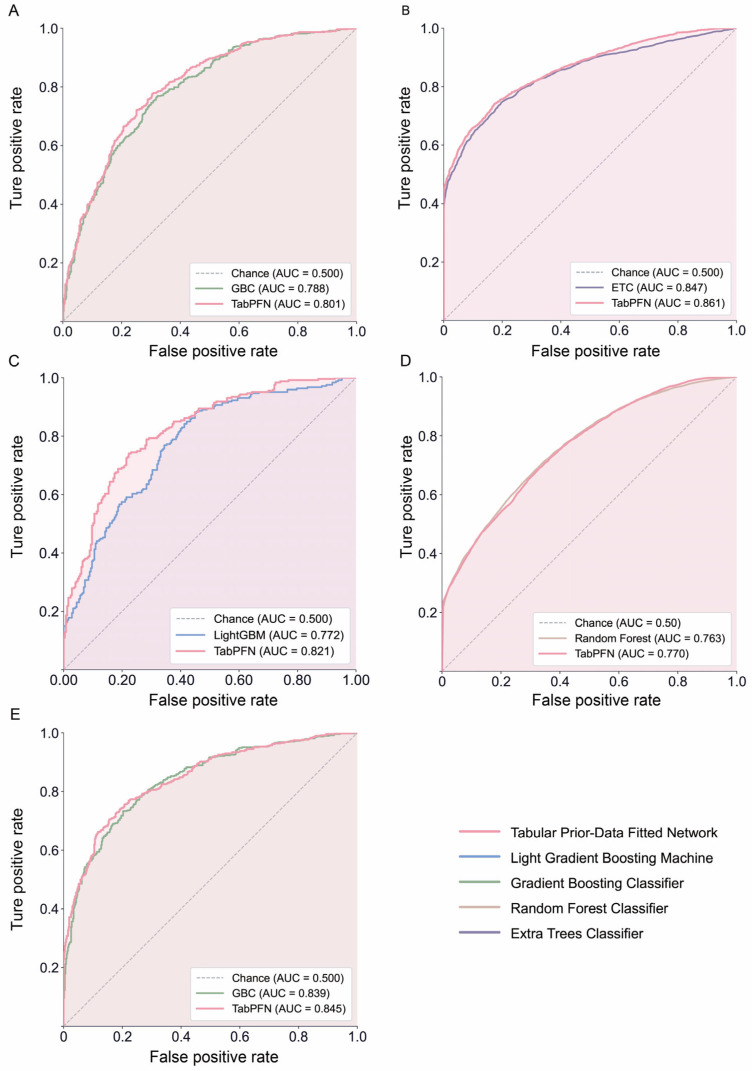
ROC curves from a single independent test set evaluation for TabPFN and the optimal machine learning models across five health states. (**A**) Disability; (**B**) Pain; (**C**) Cognitive Impairment; (**D**) Hearing Impairment; (**E**) Depression.

**Figure 4:**
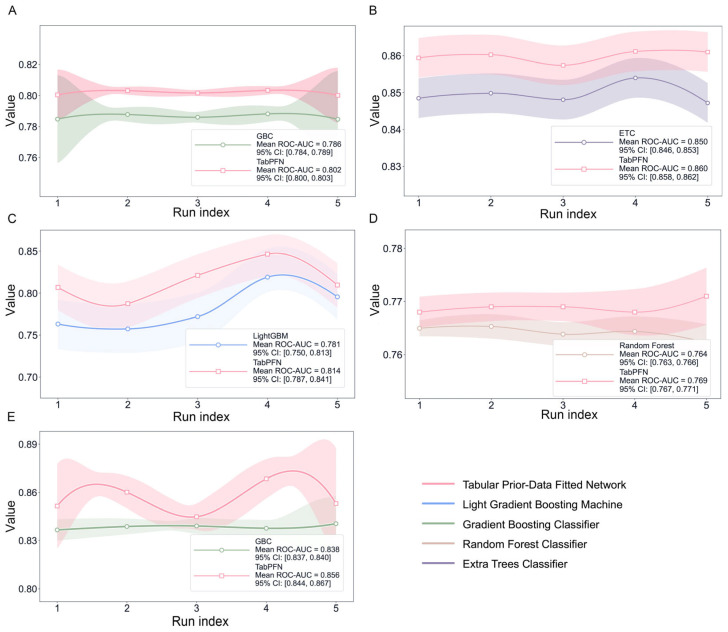
ROC confidence interval comparison between TabPFN and the optimal machine learning models across five repeated experiments for five health states. (**A**) Disability; (**B**) Pain; (**C**) Cognitive Impairment; (**D**) Hearing Impairment; (**E**) Depression.

Subsequently, the best-performing machine learning algorithm for each health outcome was compared with TabPFN on the independent test set. Performance was evaluated using Accuracy, AUC, Recall, Specificity, Precision, F1-score, Kappa, as well as the calibration metrics Brier score and Expected Calibration Error (ECE) (Refer to Table 2). In addition, the ROC curves and corresponding confidence intervals for TabPFN and the best-performing machine learning algorithm for each health outcome are presented in Figure 3 and Figure 4. It should be noted that the ROC curves in Figure 3 were generated from a single independent test, and the AUC values shown in the figure were obtained from that test. In contrast, Figure 4 presents the mean AUC and its 95% confidence interval based on five repeated experiments. As shown in Figure 3 and Figure 4, the AUC values of TabPFN for predicting disability, pain, cognitive impairment, hearing, and depression were 0.802 (95% CI: 0.800–0.803), 0.860 (95% CI: 0.858–0.862), 0.814 (95% CI: 0.787–0.841), 0.769 (95% CI: 0.767–0.771), and 0.856 (95% CI: 0.844–0.867), respectively. These values were consistently higher than those achieved by the corresponding best-performing machine learning models, namely GBC (AUC = 0.786, 95% CI: 0.784–0.789), ETC (AUC = 0.850, 95% CI: 0.846–0.853), LightGBM (AUC = 0.781, 95% CI: 0.750–0.813), RF (AUC = 0.764, 95% CI: 0.763–0.766), and GBC (AUC = 0.838, 95% CI: 0.837–0.840), respectively.

To benchmark our findings against previous studies, we additionally compared the predictive performance of TabPFN with published models for disability and hearing impairment prediction. Previous research reported an AUC of 0.724 (95% CI: 0.676–0.771) for disability prediction using XGBoost, whereas a gradient boosting machine (GBM) model applied to hearing prediction showed notable overfitting, with an AUC of 0.887 on the training set but dropping to 0.678 on the test set. To ensure a fair comparison, we replicated and evaluated the above benchmark models on a unified dataset. Results showed that XGBoost achieved an AUC of 0.7646 (95% CI: 0.7571–0.7721) on this dataset, and the GBM model achieved an AUC of 0.742 (95% CI: 0.742–0.743). In contrast, the optimal prediction model constructed using TabPFN in this study demonstrated more robust predictive performance.

Overall, these results indicate that the machine learning models demonstrated competitive performance in predicting disability, pain, cognitive impairment, hearing impairment, and depression. However, compared with the best-performing conventional machine learning model for each health outcome, TabPFN consistently achieved higher AUC values across all five prediction tasks and generally showed superior performance in terms of Accuracy, F1-score, Kappa, and MCC, demonstrating the best overall predictive performance.

### 3.3. SHAP for Model Interpretation

**Figure 5:**
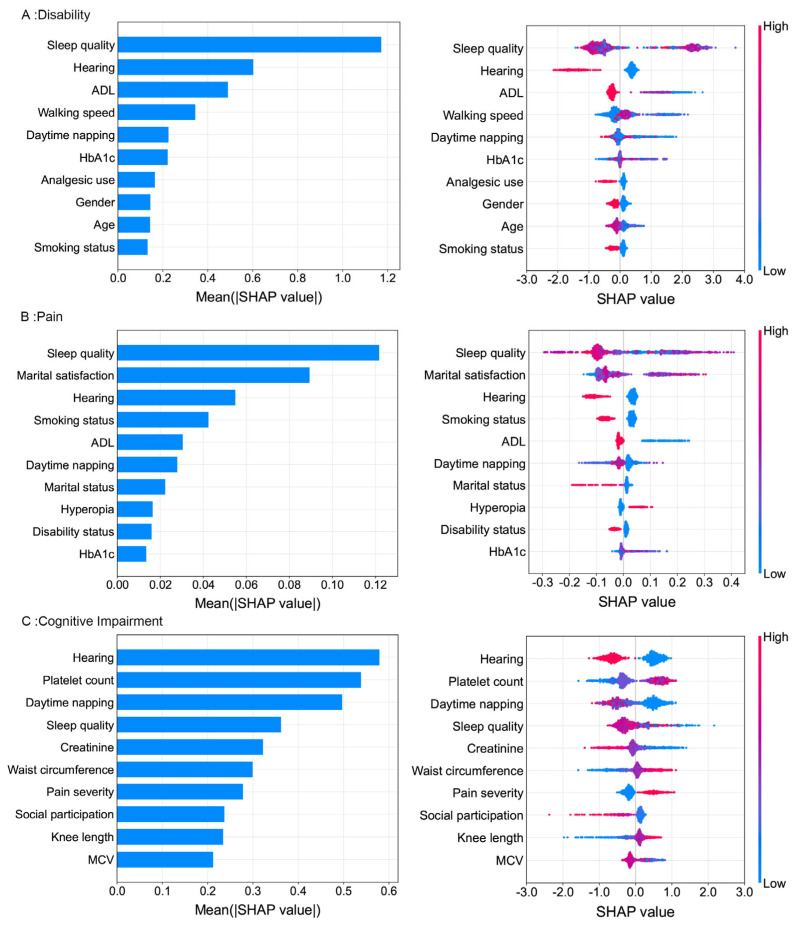

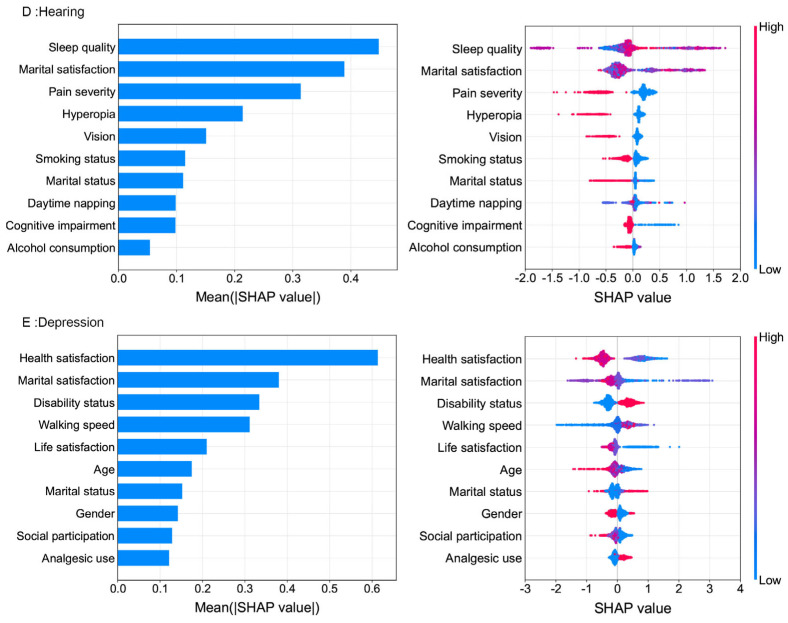
Bar charts of average SHAP values and summary plots from the optimal algorithm model. Utilizing the SHAP model to predict health states related to disability, pain, cognitive impairment, hearing loss, and depression. (**A**) Disability; (**B**) Pain; (**C**) Cognitive Impairment; (**D**) Hearing; (**E**) Depression.

To improve model interpretability, Shapley Additive Explanations (SHAP) were applied to quantify the contribution of individual features to model predictions. Feature importance was evaluated using mean absolute SHAP values, while SHAP summary plots were generated to visualize the distribution and direction of feature effects across the study population. The bar chart displays the average absolute SHAP values for each feature, reflecting their global influence on the model’s output. The SHAP summary plot provides a comprehensive view of the distribution of SHAP values for each feature and their overall impact on the dataset (Figure 5).

For disability prediction, the most significant contributory factors were “Sleep quality”, “Hearing”, “ADL”, Walking speed, and Daytime napping. Specifically, the absolute SHAP value for “Sleep quality” was the highest, indicating that this attribute is the most critical predictor for the model in forecasting disability (Figure 5A). For the prediction of pain in the elderly, SHAP interpretation analysis showed that “Sleep quality”, “Marital satisfaction,” and “Hearing” were the most influential variables. Notably, “Sleep quality” accounted for the largest contribution among all variables, exhibiting the broadest distribution, which underscores its significant effect on the prediction of pain in the model (Figure 5B). In predicting the risk of cognitive impairment in the elderly, SHAP analysis revealed that “Hearing”, “Platelet count (PLT)” and “Daytime napping” were key variables. An increase in the value of “Hearing” was associated with a reduction in the risk of cognitive impairment, suggesting that good hearing may help slow the progression of cognitive decline (Figure 5C). For hearing prediction, “Sleep quality”, “Marital satisfaction” and “Pain severity” were identified as the leading predictors (Figure 5D). In the prediction of depression among the elderly, SHAP analysis indicated that “Health satisfaction”, “Marital satisfaction” and “Disability status” were the variables that contributed most significantly to the model (Figure 5E).

Overall, the SHAP analysis identified distinct but partially overlapping predictors across the five health outcomes. These results demonstrate that the TabPFN model effectively captures a variety of important features, significantly improving its predictive performance.

## 4. Discussion

This study is based on data from the China Health and Retirement Longitudinal Study (CHARLS). It employs machine learning, deep learning, data balancing strategies, and feature selection methods to comprehensively assess disability, pain, cognitive impairment, hearing, and depression among the elderly population, and establishes predictive models. The results indicate that the TabPFN model achieved optimal performance with AUC values of 0.802 for disability, 0.860 for pain, 0.814 for cognitive impairment, 0.769 for hearing, and 0.856 for depression.

### 4.1. Advantages of TabPFN

For disability prediction, the TabPFN-based prediction model achieved an AUC of 0.802 (95% CI: 0.800–0.803), outperforming the conventional XGBoost model (AUC = 0.724, 95% CI: 0.676–0.771) as well as the logistic regression models commonly used in previous studies [9,10,17]. For pain prediction, the TabPFN model demonstrated excellent performance, with an AUC of 0.860 (95% CI: 0.858–0.862). Previous study on pain prediction have primarily focused on specific pain sites, such as knee pain and low back pain, and have mainly relied on conventional statistical methods [18]. For cognitive impairment prediction, the TabPFN model achieved an AUC of 0.814 (95% CI: 0.787–0.841), demonstrating higher predictive accuracy than conventional statistical and survival analysis methods and outperforming the CNN-BiLSTM-Attention model (AUC = 0.73) reported in a previous study [19]. For hearing impairment prediction, the TabPFN model achieved an AUC of 0.769 (95% CI: 0.767–0.771). Compared with the previously reported GBM model (training AUC = 0.887 vs. test AUC = 0.678), the TabPFN model did not exhibit overfitting [20]. In addition, the GBM model achieved an AUC of 0.742 (95% CI: 0.742–0.743) on the present dataset, which was lower than that of TabPFN. Previous studies on depression prediction in older adults are relatively abundant. In the present study, the TabPFN prediction model achieved an AUC of 0.856 (95% CI: 0.844–0.867), outperforming the conventional logistic regression model (AUC = 0.823) reported previously [21].

Overall, the findings of this study further support the application of machine learning methods for health risk prediction in middle-aged and older adults and demonstrate that TabPFN achieves favorable predictive performance for disability, pain, cognitive impairment, hearing, and depression. This performance may be attributed to the Prior-Data-Fitted learning mechanism of TabPFN, which enables the model to effectively learn prior knowledge from complex structured data while capturing nonlinear relationships and higher-order interactions among variables, thereby achieving stable and efficient predictive performance with minimal parameter tuning. In contrast, conventional machine learning algorithms still have certain limitations when handling complex health data. Logistic regression is based on the assumption of linear relationships and is therefore limited in its ability to capture the complex nonlinear associations among health-related factors. Although tree-based models, including Random Forest, XGBoost, Gradient Boosting Machine (GBM), and Light Gradient Boosting Machine (LightGBM), are capable of modeling nonlinear relationships to a certain extent, they rely primarily on iterative decision tree construction and remain limited in modeling complex feature interactions in high-dimensional heterogeneous data. Moreover, their performance depends more heavily on the optimization of hyperparameters, such as tree depth, learning rate, subsampling ratio, regularization parameters, and the number of iterations.

From an application perspective, the combination of TabPFN and SHapley Additive exPlanations (SHAP) not only enables accurate prediction of multiple health outcomes in middle-aged and older adults but also identifies the key risk factors influencing model predictions, thereby providing a scientific basis for early screening of high-risk populations, personalized health management, and targeted interventions. Moreover, with its minimal requirement for parameter tuning, strong generalization ability, and ease of deployment, the proposed approach has considerable potential for application in community health services, primary healthcare settings, and public health management. It may also support the optimization of health resource allocation, facilitate clinical decision-making, and promote the transition of health management for middle-aged and older adults from disease treatment to risk prevention. Therefore, the findings of this study suggest that TabPFN has promising application prospects for health risk prediction in middle-aged and older adults.

### 4.2. SHAP Interpretation of Key Predictors

The SHAP analysis identified the key predictors for multiple health outcomes prediction models, and the findings were generally consistent with those of previous studies. For the disability prediction model, sleep quality, hearing status, and activities of daily living (ADL) were identified as the most important predictors. Previous studies have reported a significant nonlinear relationship between sleep duration and ADL disability [22]. Individuals with better hearing have a lower risk of disability, which is consistent with findings from several geriatric studies. Chen et al. reported that hearing impairment is significantly associated with physical function decline and an increased risk of disability among older adults [23]. As a criterion for disability assessment, activities of daily living also played a key role in the prediction model. This finding is consistent with the emphasis on activities of daily living in the Chinese Standards for Physical Disability Assessment. Other variables, including walking speed, daytime napping, and HbA1c, also contributed to the disability prediction model [24].

In the prediction of pain, Sleep quality, Marital satisfaction, and Hearing were identified as the most significant contributors. Specifically, insufficient sleep was causally linked to exacerbated pain, while sufficient sleep (>6 h) could reduce pain sensitivity and the risk of chronic pain [25,26]. Higher marital satisfaction corresponded to negative SHAP values, indicating that greater marital satisfaction can lower pain risk; this is consistent with findings regarding marital satisfaction’s impact on pain severity, physical function impairment, and depression [27]. The distribution of lower hearing values in the positive SHAP region is supported by animal studies indicating that noise-induced hearing loss significantly increases pain sensitivity, suggesting an association between hearing and increased pain risk [28]. Moreover, higher smoking status correlated with negative SHAP values; a study has shown that among e-cigarette users, those using nicotine-containing e-cigarettes have significantly higher pain tolerance in cold-press tests compared to those using non-nicotine e-cigarettes [29].

In the prediction of cognitive impairment among the elderly, Hearing, Platelet count (PLT), and Daytime Napping as key variables. Hearing loss has been associated with cognitive impairment and dementia, suggesting that auditory difficulties may reflect a decline in cognitive function and an increased risk for dementia, a discovery supported by existing literature [30]. Additionally, hearing loss is recognized as an independent risk factor for cognitive decline, in line with epidemiological evidence [31,32]. An increase in PLT has been correlated with an elevated risk of cognitive impairment, which may be related to inflammatory responses or vascular factors [33]. A study has indicated that individuals with a heightened platelet response in later life face a greater risk of dementia, and platelet phenotype may be linked to dementia incidence, supporting our findings [34]. The lower distribution of the Nap variable within the positive range indicates that shorter nap durations may relate to cognitive impairment. Previous research has shown that habitual daytime napping reduces the risk of cognitive impairment and sarcopenia [35], further corroborating our findings. Moreover, lower values of Sleep quality were associated with an increased risk of cognitive impairment; studies reveal that sleeping less than the recommended duration, without experiencing daytime sleepiness, heightens the risks for brain health and cognitive function decline [36].

Concerning hearing prediction among the elderly, Sleep quality, Marital satisfaction, and Pain severity were critical variables. Literature supports a significant correlation between poor sleep quality (e.g., difficulty falling asleep, snoring, daytime sleepiness) and an increased risk of hearing loss [37]. Additionally, sleeping ≥8 h per night is associated with a 29% reduction in the risk of high-frequency hearing loss [38]. Existing research has also indicated a relationship between severe hearing loss and lower marital satisfaction [39]. In our study on pain prediction, we observed that higher Hearing scores correspond to a reduced risk of pain. Recent animal experiments provide evidence suggesting a link between hearing impairment and an increased risk of pain. This correlation was reaffirmed in the SHAP analysis for hearing prediction [28]. Notably, highvalues of Hyperopia and Vision in the SHAP analysis exhibited negative SHAP values, indicating that refractive errors are related to hearing impairment. According to research, 22% of community-dwelling older adults aged over 71 experience dual sensory impairments, with rates rising to 59% in those over 90 years old [40], consistent with our findings.

Research on predicting depression in older adults is extensive. This study found that health satisfaction, marital satisfaction, and disability status were the most significant contributors to the model. Specifically, health satisfaction had the most pronounced impact on predictions. Numerous studies have shown that depression affects self-rated health by lowering subjective well-being, thereby indirectly decreasing life satisfaction [41,42,43]. Marital satisfaction also demonstrated a strong influence, illustrating a significant negative correlation between an individual’s marital satisfaction and both their own and their spouse’s levels of depression. This association has been validated across various populations (newlyweds, older adults, pregnant women) and different countries (China, USA) [44]. Moreover, this study identified that disability increases the risk of depression. Research based on NHANES data indicates a strong correlation between disability in American adults and depressive symptoms (adjusted OR = 7.82; 95% CI: 6.27–9.75), and multiple studies consistently show that the prevalence of depression is significantly higher in individuals with disabilities compared to the general population [45], supporting the findings of this study.

Across the SHAP analyses of the five health outcomes prediction models, sleep quality and daytime napping consistently ranked among the most important features in four models. Previous studies have shown that poor sleep quality or circadian rhythm disruption may affect cognitive function, pain perception, physical function, and sensory function [46]. Hearing status, marital satisfaction, smoking status, and marital status were identified as important predictors in three health outcomes prediction models. Previous evidence suggests that hearing loss not only impairs social communication but may also contribute to social isolation, reduced physical activity, and diminished cognitive reserve, thereby making it an important predictor across multiple health outcomes models [47,48]. Marital satisfaction and marital status were repeatedly identified as important features in multiple models, and previous studies have reported that marriage may influence mental health and social relationships, thereby affecting the health of older adults [49]. In addition, long-term smoking has been shown to increase the risk of various chronic diseases and functional impairments [50]. Meanwhile, activities of daily living (ADL), walking speed, HbA1c, and social participation were identified as important predictors in two models. ADL and walking speed reflect overall functional reserve and physical performance in older adults [50,51]. As an indicator of long-term glycemic control, HbA1c may influence different health outcomes through mechanisms involving vascular injury, inflammatory responses, and oxidative stress [52,53]. Therefore, the consistent importance of these variables across multiple health outcomes prediction models is clinically plausible.

SHAP analysis reflects the relative contribution of each variable to the model predictions rather than causal relationships between variables and health outcomes. Because this study was based on observational data, potential confounding, reverse causality, and shared pathological mechanisms may exist among the variables. Therefore, the findings should be interpreted as indicating the predictive value and clinical relevance of these variables rather than as evidence of direct causal relationships with the health outcomes. Further validation of the underlying causal mechanisms will require longitudinal cohort studies and intervention studies.

### 4.3. Innovations

This study simultaneously focused on five important health conditions among middle-aged and older adults, including disability, pain, cognitive impairment, hearing impairment, and depression. Prediction models were developed separately based on the CHARLS cohort, and the emerging tabular foundation model TabPFN was introduced for systematic comparison with multiple conventional machine learning algorithms. Compared with previous studies that mainly focused on predicting a single health condition, this study evaluated the applicability and predictive performance of different machine learning algorithms in health risk prediction among middle-aged and older adults from the perspective of multiple health outcomes, providing broader evidence for the application of machine learning methods in geriatric health risk prediction.

Furthermore, this study incorporated SHAP analysis to interpret the prediction models, identifying key influencing factors associated with different health conditions and exploring potential common factors across multiple health conditions. These findings provide a new perspective for understanding the characteristics of multidimensional health risks among middle-aged and older adults and conducting comprehensive risk assessment.

### 4.4. Limitations

This study has several limitations. First, because there is currently no publicly available independent cohort with the same variable system and health outcomes definitions as CHARLS, all models were developed and validated using the CHARLS database, and external validation has not yet been performed. Therefore, the generalizability of the models requires further evaluation. Second, some variables contained missing values. Although regression imputation and mean imputation were applied during data preprocessing, these missing data may still have affected model performance. In addition, this study was primarily based on existing survey data and did not incorporate multicenter clinical data or long-term longitudinal follow-up data.

### 4.5. Future Directions

Further studies are needed to improve the generalizability and clinical applicability of the proposed models. First, because all models were developed and validated using the CHARLS database, future studies should perform external validation in multicenter, multi-regional, and multiethnic populations. Second, the prediction models in this study were developed using baseline survey data. Future studies could incorporate long-term longitudinal follow-up data to establish dynamic health risk prediction models for continuous monitoring of health outcomes changes and disease progression in middle-aged and older adults.

In addition, the SHAP analysis identified several factors that were consistently important across multiple health outcomes prediction models. Future prospective cohort studies and mechanistic investigations are needed to further explore the relationships between these common predictors and different health outcomes. Finally, given the favorable predictive performance of TabPFN across all five health outcomes prediction tasks, future studies may further investigate multi-task learning and multimodal data fusion strategies by integrating data from medical imaging, genetic information, and wearable devices to further improve predictive performance and clinical applicability, facilitate the translation of the model into a clinical decision support tool, and support precise health risk assessment and individualized management for middle-aged and older adults.

## 5. Conclusions

This study developed prediction models for five health outcomes among middle-aged and older adults—disability, pain, cognitive impairment, hearing impairment, and depression—using data from the CHARLS database, and systematically compared the predictive performance of multiple machine learning algorithms. The results demonstrated that TabPFN achieved the best performance across all five prediction tasks, exhibiting robust predictive accuracy and strong generalization ability. Benefiting from its large-scale pretraining strategy, TabPFN effectively captured complex nonlinear relationships among variables and produced reliable predictions in small- to medium-sized datasets without extensive hyperparameter tuning, thereby enhancing model reproducibility, usability, and clinical applicability. These characteristics make it particularly well suited for modeling structured clinical tabular data.

Based on SHAP interpretability analysis, this study further identified the key factors associated with different health outcomes, improving the interpretability of the prediction models and suggesting that different health conditions may share common risk factors. These findings provide a new perspective for the joint risk assessment of multiple health outcomes. Overall, this study validated the application value of TabPFN in predicting multiple health outcomes among middle-aged and older adults, providing a methodological basis for the application of artificial intelligence in early health risk identification, precise risk assessment, and personalized health management, while also offering scientific evidence to support community health screening, primary healthcare management, and public health decision-making.

## Figures and Tables

**Table 1:** Baseline characteristics of predictors for disability.

	Total (*N* = 20,967)	Non-Disability (*N* = 18,050)	Disability (*N* = 2917)	*p*.Overall
Waist circumference	85.8 (18.13)	85.81 (17.71)	85.78 (20.56)	0.951
Systolic blood pressure (SBP)	132.09(61.86)	131.94 (61.52)	133.06 (63.91)	0.376
Diastolic blood pressure (DBP)	75.37 (10.37)	75.34 (10.38)	75.51 (10.28)	0.415
Pulse rate	74.12 (9.45)	74.11 (9.49)	74.17 (9.2)	0.723
Peak Expiratory Flow (PEF)	292.33 (118.32)	291.9 (117.85)	294.96(121.18)	0.205
Left hand grip strength	29.36 (45.36)	29.26 (44.26)	29.98 (51.66)	0.476
Right hand grip strength	30.64 (43.97)	30.64 (43.85)	30.62 (44.72)	0.974
Walking speed	1.68 (3.52)	1.68 (3.56)	1.65 (3.25)	0.629
BMI	23.90 (3.92)	23.89 (3.89)	23.95 (4.11)	0.513
WBC	5.89 (1.63)	5.89 (1.65)	5.93 (1.51)	0.144
Hb	13.66 (1.55)	13.66 (1.53)	13.64 (1.63)	0.502
Hct	41.35 (4.52)	41.36 (4.49)	41.29 (4.69)	0.405
MCV	91.57 (6.19)	91.58 (6.19)	91.56 (6.16)	0.87
PLT	203.62 (60.39)	203.27 (59.42)	205.76 (66.04)	0.056
TG	132.83 (74.01)	132.41 (73.35)	135.41 (77.93)	0.052
Cr	0.79 (0.23)	0.79 (0.23)	0.79 (0.21)	0.816
HDL	50.67 (9.22)	50.7 (9.19)	50.52 (9.36)	0.342
LDL	101.35 (23.14)	101.47 (23.05)	100.62 (23.68)	0.072
TC	182.62 (29.2)	182.69 (29.0)	182.16 (30.42)	0.375
GLU	100.6 (28.4)	100.47 (27.97)	101.4 (30.93)	0.126
UA	4.89 (1.13)	4.89 (1.13)	4.91 (1.14)	0.356
Cys-C	0.84 (0.19)	0.84 (0.19)	0.84 (0.24)	0.368
CRP	2.24 (4.8)	2.24 (4.75)	2.28 (5.12)	0.657
HbA1c	5.91 (0.79)	5.91 (0.78)	5.93 (0.88)	0.122
Age	68.2 (10.47)	68.19 (10.45)	68.26 (10.6)	0.731
Sleep quality	6.4 (1.86)	6.45 (1.82)	6.11 (2.04)	<0.001
Daytime napping	38.0 (43.28)	38.25 (43.34)	36.43 (42.87)	0.034
Upper Arm Length	33.38 (2.32)	33.37 (2.30)	33.42 (2.39)	0.328
Knee Length	47.59 (3.08)	47.57 (3.08)	47.72 (3.05)	0.015
Activities of daily living (ADL)	5.88 (0.6)	5.92 (0.46)	5.6 (1.1)	<0.001
Standing balance				0.356
1	20,877 (99.6%)	17,969 (99.6%)	2908 (99.7%)	
0	90 (0.4%)	81 (0.4%)	9 (0.3%)	
Gender				0.054
0	10,995 (52.4%)	9514 (52.7%)	1481 (50.8%)	
1	9972 (47.6%)	8536 (47.3%)	1436 (49.2%)	
Marital status				0.511
1	16,919 (80.7%)	14,542 (80.6%)	2377 (81.5%)	
5	2321 (11.1%)	1992 (11.0%)	329 (11.3%)	
2	1296 (6.2%)	1138 (6.3%)	158 (5.4%)	
3	56 (0.3%)	48 (0.3%)	8 (0.3%)	
4	192 (0.9%)	171 (0.9%)	21 (0.7%)	
6	157 (0.7%)	137 (0.8%)	20 (0.7%)	
7	26 (0.1%)	22 (0.1%)	4 (0.1%)	
Hearing				<0.001
0	13,708 (65.4%)	11,271 (62.4%)	2437 (83.5%)	
1	7259 (34.6%)	6779 (37.6%)	480 (16.5%)	
Smoking Status				0.105
1	8486 (40.5%)	7265 (40.2%)	1221 (41.9%)	
0	12,481 (59.5%)	10,785 (59.8%)	1696 (58.1%)	
Alcohol consumption				0.696
0	13,561 (64.7%)	11,673 (64.7%)	1888 (64.7%)	
2	5563 (26.5%)	4801 (26.6%)	762 (26.1%)	
1	1843 (8.8%)	1576 (8.7%)	267 (9.2%)	
Social participation				0.666
0	8756 (41.8%)	7565 (41.9%)	1191 (40.8%)	
3	1347 (6.4%)	1160 (6.4%)	187 (6.4%)	
2	3029 (14.4%)	2570 (14.2%)	459 (15.7%)	
1	6923 (33.0%)	5967 (33.1%)	956 (32.8%)	
4	523 (2.5%)	457 (2.5%)	66 (2.3%)	
5	242 (1.2%)	204 (1.1%)	38 (1.3%)	
6	93 (0.4%)	80 (0.4%)	13 (0.4%)	
8	11 (0.1%)	9 (0.0%)	2 (0.1%)	
7	38 (0.2%)	34 (0.2%)	4 (0.1%)	
9	5 (0.0%)	4 (0.0%)	1 (0.0%)	
Health satisfaction				0.399
2	11,185 (53.3%)	9586 (53.1%)	1599 (54.8%)	
3	4034 (19.2%)	3479 (19.3%)	555 (19.0%)	
1	3755 (17.9%)	3265 (18.1%)	490 (16.8%)	
0	1257 (6.0%)	1088 (6.0%)	169 (5.8%)	
4	736 (3.5%)	632 (3.5%)	104 (3.6%)	
Marital satisfaction				0.659
2	11,055 (52.7%)	9529 (52.8%)	1526 (52.3%)	
3	7231 (34.5%)	6216 (34.4%)	1015 (34.8%)	
1	944 (4.5%)	820 (4.5%)	124 (4.3%)	
0	364 (1.7%)	315 (1.7%)	49 (1.7%)	
4	1364 (6.5%)	1161 (6.4%)	203 (7.0%)	
6	9 (0.0%)	9 (0.0%)	0 (0.0%)	
Life satisfaction				0.444
3	7053 (33.6%)	6073 (33.6%)	980 (33.6%)	
2	10,940 (52.2%)	9441 (52.3%)	1499 (51.4%)	
1	1325 (6.3%)	1141 (6.3%)	184 (6.3%)	
4	1312 (6.3%)	1107 (6.1%)	205 (7.0%)	
0	337 (1.6%)	288 (1.6%)	49 (1.7%)	
Prostate medication				<0.001
0	20,577 (98.1%)	17,754 (98.4%)	2823 (96.8%)	
1	390 (1.9%)	296 (1.6%)	94 (3.2%)	
Antihypertensive medication				0.005
0	15,742 (75.1%)	13,613 (75.4%)	2129 (73.0%)	
1	5225 (24.9%)	4437 (24.6%)	788 (27.0%)	
Diabetes medication				0.91
0	19,602 (93.5%)	16,873 (93.5%)	2729 (93.6%)	
1	1365 (6.5%)	1177 (6.5%)	188 (6.4%)	
Cancer medication				<0.001
0	20,804 (99.2%)	17,927 (99.3%)	2877 (98.6%)	
1	163 (0.8%)	123 (0.7%)	40 (1.4%)	
Analgesic use				0.315
0	14,956 (71.3%)	12,852 (71.2%)	2104 (72.1%)	
1	6011 (28.7%)	5198 (28.8%)	813 (27.9%)	
Pain severity				<0.001
0	15,124 (72.1%)	13,453 (74.5%)	1671 (57.3%)	
1	5843 (27.9%)	4597 (25.5%)	1246 (42.7%)	
Cataract surgery				<0.001
0	20,756 (99.0%)	17,886 (99.1%)	2870 (98.4%)	
1	211 (1.0%)	164 (0.9%)	47 (1.6%)	
Glaucoma				<0.001
0	20,710 (98.8%)	17,879 (99.1%)	2831 (97.1%)	
1	257 (1.2%)	171 (0.9%)	86 (2.9%)	
Depression status				0.578
0	18,732 (89.3%)	16,115 (89.3%)	2617 (89.7%)	
1	2169 (10.3%)	1880 (10.4%)	289 (9.9%)	
2	66 (0.3%)	55 (0.3%)	11 (0.4%)	
Myopia				<0.001
0	17,208 (82.1%)	15,065 (83.5%)	2143 (73.5%)	
1	3759 (17.9%)	2985 (16.5%)	774 (26.5%)	
Hyperopia				<0.001
0	17,156 (81.8%)	15,145 (83.9%)	2011 (68.9%)	
1	3811 (18.2%)	2905 (16.1%)	906 (31.1%)	
Cognitive impairment				<0.001
1	19,738 (94.1%)	16,897 (93.6%)	2841 (97.4%)	
0	1229 (5.9%)	1153 (6.4%)	76 (2.6%)	
Disability status				<0.001
0	18,050 (86.1%)	18,050 (100.0%)	0 (0.0%)	
1	2917 (13.9%)	0 (0.0%)	2917 (100.0%)	
Hypertension				<0.001
0	19,569 (93.3%)	16,893 (93.6%)	2676 (91.7%)	
1	1398 (6.7%)	1157 (6.4%)	241 (8.3%)	
Dyslipidemia				0.049
0	20,065 (95.7%)	17,294 (95.8%)	2771 (95.0%)	
1	902 (4.3%)	756 (4.2%)	146 (5.0%)	
Diabetes mellitus				<0.001
0	20,401 (97.3%)	17,613 (97.6%)	2788 (95.6%)	
1	566 (2.7%)	437 (2.4%)	129 (4.4%)	
Malignant tumor				0.116
0	20,872 (99.5%)	17,974 (99.6%)	2898 (99.3%)	
1	95 (0.5%)	76 (0.4%)	19 (0.7%)	
Lung disease				<0.001
0	20,535 (97.9%)	17,717 (98.2%)	2818 (96.6%)	
1	432 (2.1%)	333 (1.8%)	99 (3.4%)	
Liver disease				0.273
0	20,712 (98.8%)	17,837 (98.8%)	2875 (98.6%)	
1	255 (1.2%)	213 (1.2%)	42 (1.4%)	
Heart disease				<0.001
0	20,248 (96.6%)	17,469 (96.8%)	2779 (95.3%)	
1	719 (3.4%)	581 (3.2%)	138 (4.7%)	
Stroke				<0.001
0	20,810 (99.3%)	17,963 (99.5%)	2847 (97.6%)	
1	157 (0.7%)	87 (0.5%)	70 (2.4%)	
Asthma				0.027
0	20,833 (99.4%)	17,944 (99.4%)	2889 (99.0%)	
1	134 (0.6%)	106 (0.6%)	28 (1.0%)	
Assistive Device Use				<0.001
0	19,460 (92.8%)	17,086 (94.7%)	2374 (81.4%)	
1	1507 (7.2%)	964 (5.3%)	543 (18.6%)	
Edentulism/Complete Tooth Loss				<0.001
0	20,150 (96.1%)	17,410 (96.5%)	2740 (93.9%)	
1	817 (3.9%)	640 (3.5%)	177 (6.1%)	
Kidney disease				<0.001
0	20,582 (98.2%)	17,746 (98.3%)	2836 (97.2%)	
1	385 (1.8%)	304 (1.7%)	81 (2.8%)	
Stomach disease				0.037
0	20,311 (96.9%)	17,504 (97.0%)	2807 (96.2%)	
1	656 (3.1%)	546 (3.0%)	110 (3.8%)	
Affective disorder				<0.001
0	20,884 (99.6%)	17,999 (99.7%)	2885 (98.9%)	
1	83 (0.4%)	51 (0.3%)	32 (1.1%)	
Memory-related disease				<0.001
0	20,807 (99.2%)	17,950 (99.4%)	2857 (97.9%)	
1	160 (0.8%)	100 (0.6%)	60 (2.1%)	
Arthritis				0.01
0	20,307 (96.9%)	17,505 (97.0%)	2802 (96.1%)	
1	660 (3.1%)	545 (3.0%)	115 (3.9%)	

**Table 2:** Test-set performance metrics (mean ± SD) of TabPFN and the best-performing machine learning models across five health outcomes.

Health Outcomes	Model	Accuracy	AUC	Recall	Specificity	Precision	F1	Kappa	Brier	ECE
Disability	TabPFN	0.734 ± 0.003	0.802 ± 0.001	0.731 ± 0.002	0.738 ± 0.005	0.736 ± 0.004	0.734 ± 0.003	0.469 ± 0.006	0.183 ± 0.002	0.030 ± 0.008
	GBC	0.716 ± 0.007	0.786 ± 0.002	0.716 ± 0.007	0.715 ± 0.011	0.716 ± 0.008	0.716 ± 0.006	0.431 ± 0.013	0.188 ± 0.001	0.031 ± 0.008
Pain	TabPFN	0.779 ± 0.002	0.860 ± 0.002	0.678 ± 0.011	0.879 ± 0.010	0.848 ± 0.009	0.754 ± 0.004	0.557 ± 0.003	0.151 ± 0.001	0.020 ± 0.004
	ETC	0.775 ± 0.004	0.850 ± 0.003	0.715 ± 0.004	0.835 ± 0.005	0.812 ± 0.005	0.761 ± 0.004	0.550 ± 0.008	0.151 ± 0.000	0.044 ± 0.001
Cognitive Impairment	TabPFN	0.731 ± 0.021	0.814 ± 0.022	0.713 ± 0.044	0.750 ± 0.017	0.740 ± 0.014	0.726 ± 0.027	0.462 ± 0.042	0.160 ± 0.000	0.032 ± 0.004
	LGBM	0.696 ± 0.030	0.781 ± 0.026	0.712 ± 0.044	0.680 ± 0.041	0.690 ± 0.030	0.700 ± 0.032	0.392 ± 0.060	0.171 ± 0.000	0.059 ± 0.000
Hearing Impairment	TabPFN	0.679 ± 0.005	0.769 ± 0.001	0.689 ± 0.012	0.669 ± 0.009	0.676 ± 0.005	0.682 ± 0.006	0.358 ± 0.009	0.197 ± 0.001	0.028 ± 0.010
	RF	0.683 ± 0.003	0.764 ± 0.001	0.645 ± 0.004	0.720 ± 0.004	0.698 ± 0.003	0.670 ± 0.003	0.365 ± 0.005	0.197 ± 0.000	0.038 ± 0.002
Depression	TabPFN	0.782 ± 0.011	0.856 ± 0.009	0.759 ± 0.013	0.806 ± 0.021	0.796 ± 0.017	0.777 ± 0.010	0.565 ± 0.022	0.154 ± 0.005	0.037 ± 0.004
	GBC	0.767 ± 0.005	0.838 ± 0.001	0.745 ± 0.011	0.790 ± 0.006	0.780 ± 0.004	0.762 ± 0.007	0.535 ± 0.011	0.164 ± 0.001	0.035 ± 0.007

## Data Availability

The data for this study are all from the China Health and Retirement Longitudinal Study database, available at: https://charls.pku.edu.cn/.

## Supplementary Materials

The following supporting information can be downloaded at: https://www.mdpi.com/article/10.3390/healthcare14142223/s1, Table S1. Baseline characteristics of predictors for pain. Table S2. Baseline characteristics of predictors for cognitive impairment. Table S3. Baseline characteristics of predictors for hearing. Table S4. Baseline characteristics of predictors for depression.

## Abbreviations

The following abbreviations are used in this manuscript:

CHARLS	China Health and Retirement Longitudinal Study
MRMR	Minimum Redundancy Maximum Relevance
IFS	Incremental Feature Selection
ROC-AUC	Receiver Operating Characteristic—Area Under the Curve
TabPFN	Tabular Prior-data Fitted Network
Ridge	Ridge Regression
GBC	Gradient Boosting Classifier
LightGBM	Light Gradient Boosting Machine
LDA	Linear Discriminant Analysis
LR	Logistic Regression
RF	Random Forest
AdaBoost	Adaptive Boosting
ExtraTrees	Extremely Randomized Trees
NB	Naive Bayes
DT	Decision Tree
SVM (linear)	Support Vector Machine (linear kernel)
KNN	K-Nearest Neighbors
QDA	Quadratic Discriminant Analysis
Dummy	Dummy Classifier
XGBoost	eXtreme Gradient Boosting
Shapley	Shapley Value
ADL	Activities of Daily Living
Logistic	Logistic Regression
CES-D	Center for Epidemiologic Studies Depression Scale
WBC	White Blood Cell
Hb	Hemoglobin
Hct	Hematocrit
MCV	Mean Corpuscular Volume
PLT	Platelet
TG	Triglyceride
Cr	Creatinine
HDL	High-Density Lipoprotein
LDL	Low-Density Lipoprotein
TC	Total Cholesterol
GLU	Glucose
UA	Uric Acid
Cys_c	Cystatin C
CRP	C-reactive Protein
HbA1c	Hemoglobin A1c
