# Supplementary material for: Machine Learning-Based Multidimensional Health Decline Prediction Framework: Data-Driven Modeling for the Middle-Aged and Elderly Population
Source: Healthcare (Basel). 2026 Jul 22;14(14):2223. doi: 10.3390/healthcare14142223 (PMC13409845; doi:10.3390/healthcare14142223)
Supplement: Supplementary file 1 [file healthcare-14-02223-s001.zip › healthcare-4395276-supplementary.pdf]

Table S1. Baseline characteristics of predictors for pain

	Total (N=20967)	Non-pain (N=15124)	pain (N=5843)	p.overall
Waist circumference	85.8 (18.13)	85.81 (18.7)	85.78 (16.57)	0.899
Systolic blood pressure (SBP)	132.09 (61.86)	132.06 (62.44)	132.19 (60.32)	0.89
Diastolic blood pressure (DBP)	75.37 (10.37)	75.32 (10.4)	75.49 (10.28)	0.303
Pulse rate	74.12 (9.45)	74.15 (9.46)	74.02 (9.43)	0.369
Peak Expiratory Flow (PEF)	292.33 (118.32)	291.66 (117.14)	294.06 (121.32)	0.196
Left hand grip strength	29.36 (45.36)	29.29 (44.66)	29.54 (47.13)	0.721
Right hand grip strength	30.64 (43.97)	30.5 (42.61)	31.01 (47.31)	0.465
Walking speed	1.68 (3.52)	1.69 (3.87)	1.63 (2.41)	0.161
BMI	23.90(3.92)	23.86(3.89)	24.00(4.00)	0.049
WBC	5.89 (1.63)	5.89 (1.69)	5.91 (1.46)	0.447
Hb	13.66 (1.55)	13.64 (1.55)	13.71 (1.55)	0.001
Hct	41.35 (4.52)	41.3 (4.52)	41.5 (4.51)	0.004
MCV	91.57 (6.19)	91.52 (6.25)	91.71 (6.01)	0.046
PLT	203.62 (60.39)	203.65 (60.48)	203.54 (60.17)	0.902
TG	132.83 (74.01)	132.9 (74.96)	132.64 (71.51)	0.813
Cr	0.79 (0.23)	0.79 (0.24)	0.78 (0.2)	0.157
HDL	50.67 (9.22)	50.65 (9.23)	50.74 (9.19)	0.531
LDL	101.35 (23.14)	101.21 (23.1)	101.71 (23.24)	0.159
TC	182.62 (29.2)	182.54 (29.22)	182.81 (29.16)	0.561
GLU	100.6 (28.4)	100.68 (28.48)	100.38 (28.19)	0.493
UA	4.89 (1.13)	4.89 (1.13)	4.89 (1.12)	0.968
Cys-C	0.84 (0.19)	0.84 (0.19)	0.84 (0.2)	0.343
CRP	2.24 (4.8)	2.28 (4.96)	2.16 (4.38)	0.109
HbA1c	5.91 (0.79)	5.91 (0.8)	5.9 (0.77)	0.468
Age	68.2 (10.47)	68.2 (10.52)	68.19 (10.37)	0.986
Sleep quality	6.4 (1.86)	6.62 (1.7)	5.85 (2.1)	<0.001
Daytime napping	38.0 (43.28)	39.28 (43.06)	34.67 (43.67)	<0.001
Activities of daily living (ADL)	5.88 (0.6)	5.91 (0.55)	5.8 (0.71)	<0.001
Upper Arm Length	33.38(2.32)	33.39(2.28)	33.35(2.41)	0.241
Knee Length	47.59(3.08)	47.59(3.08)	47.59(3.06)	0.863
Asthma				<0.001
0	20833 (99.4%)	15055 (99.5%)	5778 (98.9%)	
1	134 (0.6%)	69 (0.5%)	65 (1.1%)	
Assistive Device Use				<0.001
0	19460 (92.8%)	14292 (94.5%)	5168 (88.4%)	

1	1507 (7.2%)	832 (5.5%)	675 (11.6%)	
Edentulism / Complete Tooth Loss				<0.001
0	20150 (96.1%)	14588 (96.5%)	5562 (95.2%)	
1	817 (3.9%)	536 (3.5%)	281 (4.8%)	
Standing balance				0.28
1	20877 (99.6%)	15054 (99.5%)	5823 (99.7%)	
0	90 (0.4%)	70 (0.5%)	20 (0.3%)	
Gender				0.26
0	10995 (52.4%)	7968 (52.7%)	3027 (51.8%)	
1	9972 (47.6%)	7156 (47.3%)	2816 (48.2%)	
Marital status				0.853
1	16919 (80.7%)	12229 (80.9%)	4690 (80.3%)	
5	2321 (11.1%)	1659 (11.0%)	662 (11.3%)	
2	1296 (6.2%)	921 (6.1%)	375 (6.4%)	
3	56 (0.3%)	43 (0.3%)	13 (0.2%)	
4	192 (0.9%)	136 (0.9%)	56 (1.0%)	
6	157 (0.7%)	117 (0.8%)	40 (0.7%)	
7	26 (0.1%)	19 (0.1%)	7 (0.1%)	
Hearing				0.399
0	13561 (64.7%)	9807 (64.8%)	3754 (64.2%)	
1	5563 (26.5%)	4012 (26.5%)	1551 (26.5%)	
Smoking Status	1843 (8.8%)	1305 (8.6%)	538 (9.2%)	
1				0.282
0	8756 (41.8%)	6311 (41.7%)	2445 (41.8%)	
Alcohol consumption	1347 (6.4%)	977 (6.5%)	370 (6.3%)	
0	3029 (14.4%)	2143 (14.2%)	886 (15.2%)	
2	6923 (33.0%)	5047 (33.4%)	1876 (32.1%)	
1	523 (2.5%)	372 (2.5%)	151 (2.6%)	
Social participation	242 (1.2%)	161 (1.1%)	81 (1.4%)	
0	93 (0.4%)	71 (0.5%)	22 (0.4%)	
3	11 (0.1%)	9 (0.1%)	2 (0.0%)	
2	38 (0.2%)	29 (0.2%)	9 (0.2%)	
1	5 (0.0%)	4 (0.0%)	1 (0.0%)	
4				0.604
5	11185 (53.3%)	8094 (53.5%)	3091 (52.9%)	
6	4034 (19.2%)	2875 (19.0%)	1159 (19.8%)	
8	3755 (17.9%)	2712 (17.9%)	1043 (17.9%)	
7	1257 (6.0%)	919 (6.1%)	338 (5.8%)	
9	736 (3.5%)	524 (3.5%)	212 (3.6%)	
Health satisfaction				0.272
2	11055 (52.7%)	8018 (53.0%)	3037 (52.0%)	
3	7231 (34.5%)	5159 (34.1%)	2072 (35.5%)	
1	944 (4.5%)	694 (4.6%)	250 (4.3%)	
0	364 (1.7%)	271 (1.8%)	93 (1.6%)	
4	1364 (6.5%)	977 (6.5%)	387 (6.6%)	

Marital satisfaction	9 (0.0%)	5 (0.0%)	4 (0.1%)	
2				0.493
3	7053 (33.6%)	5034 (33.3%)	2019 (34.6%)	
1	10940 (52.2%)	7927 (52.4%)	3013 (51.6%)	
0	1325 (6.3%)	960 (6.3%)	365 (6.2%)	
4	1312 (6.3%)	954 (6.3%)	358 (6.1%)	
6	337 (1.6%)	249 (1.6%)	88 (1.5%)	
Life satisfaction				<0.001
3	20577 (98.1%)	14896 (98.5%)	5681 (97.2%)	
2	390 (1.9%)	228 (1.5%)	162 (2.8%)	
1				0.365
4	15742 (75.1%)	11381 (75.3%)	4361 (74.6%)	
0	5225 (24.9%)	3743 (24.7%)	1482 (25.4%)	
Prostate medication				0.76
0	19602 (93.5%)	14134 (93.5%)	5468 (93.6%)	
1	1365 (6.5%)	990 (6.5%)	375 (6.4%)	
Antihypertensive medication				0.716
0	20804 (99.2%)	15009 (99.2%)	5795 (99.2%)	
1	163 (0.8%)	115 (0.8%)	48 (0.8%)	
Diabetes medication				0.855
0	14956 (71.3%)	10794 (71.4%)	4162 (71.2%)	
1	6011 (28.7%)	4330 (28.6%)	1681 (28.8%)	
Cancer medication				<0.001
0	19738 (94.1%)	14091 (93.2%)	5647 (96.6%)	
1	1229 (5.9%)	1033 (6.8%)	196 (3.4%)	
Analgesic use				0.035
0	20756 (99.0%)	14986 (99.1%)	5770 (98.8%)	
1	211 (1.0%)	138 (0.9%)	73 (1.2%)	
Pain severity				<0.001
0	20710 (98.8%)	14983 (99.1%)	5727 (98.0%)	
1	257 (1.2%)	141 (0.9%)	116 (2.0%)	
Cataract surgery				<0.001
0	17208 (82.1%)	13091 (86.6%)	4117 (70.5%)	
1	3759 (17.9%)	2033 (13.4%)	1726 (29.5%)	
Glaucoma				<0.001
0	17156 (81.8%)	13195 (87.2%)	3961 (67.8%)	
1	3811 (18.2%)	1929 (12.8%)	1882 (32.2%)	
Depression status				0.876
0	18732 (89.3%)	13522 (89.4%)	5210 (89.2%)	
1	2169 (10.3%)	1555 (10.3%)	614 (10.5%)	
2	66 (0.3%)	47 (0.3%)	19 (0.3%)	
Myopia				<0.001
0	18050 (86.1%)	13453 (89.0%)	4597 (78.7%)	
1	2917 (13.9%)	1671 (11.0%)	1246 (21.3%)	
Hyperopia				<0.001

0	19569 (93.3%)	14180 (93.8%)	5389 (92.2%)	
1	1398 (6.7%)	944 (6.2%)	454 (7.8%)	
Cognitive impairment				<0.001
1	20065 (95.7%)	14568 (96.3%)	5497 (94.1%)	
0	902 (4.3%)	556 (3.7%)	346 (5.9%)	
Disability status				<0.001
0	20401 (97.3%)	14764 (97.6%)	5637 (96.5%)	
1	566 (2.7%)	360 (2.4%)	206 (3.5%)	
Hypertension				0.356
0	20872 (99.5%)	15060 (99.6%)	5812 (99.5%)	
1	95 (0.5%)	64 (0.4%)	31 (0.5%)	
Dyslipidemia				<0.001
0	20535 (97.9%)	14890 (98.5%)	5645 (96.6%)	
1	432 (2.1%)	234 (1.5%)	198 (3.4%)	
Diabetes mellitus				<0.001
0	20712 (98.8%)	14971 (99.0%)	5741 (98.3%)	
1	255 (1.2%)	153 (1.0%)	102 (1.7%)	
Malignant tumor				<0.001
0	20248 (96.6%)	14695 (97.2%)	5553 (95.0%)	
1	719 (3.4%)	429 (2.8%)	290 (5.0%)	
Lung disease				0.036
0	20810 (99.3%)	15023 (99.3%)	5787 (99.0%)	
1	157 (0.7%)	101 (0.7%)	56 (1.0%)	
Liver disease				<0.001
0	20582 (98.2%)	14916 (98.6%)	5666 (97.0%)	
1	385 (1.8%)	208 (1.4%)	177 (3.0%)	
Heart disease				<0.001
0	20311 (96.9%)	14751 (97.5%)	5560 (95.2%)	
1	656 (3.1%)	373 (2.5%)	283 (4.8%)	
Stroke				<0.001
0	20884 (99.6%)	15084 (99.7%)	5800 (99.3%)	
1	83 (0.4%)	40 (0.3%)	43 (0.7%)	
Kidney disease				<0.001
0	20807 (99.2%)	15037 (99.4%)	5770 (98.8%)	
1	160 (0.8%)	87 (0.6%)	73 (1.2%)	
Stomach disease				<0.001
0	20307 (96.9%)	14763 (97.6%)	5544 (94.9%)	
1	660 (3.1%)	361 (2.4%)	299 (5.1%)	
Affective disorder				<0.001
0	20833 (99.4%)	15055 (99.5%)	5778 (98.9%)	
1	134 (0.6%)	69 (0.5%)	65 (1.1%)	
Memory-related disease				<0.001
0	19460 (92.8%)	14292 (94.5%)	5168 (88.4%)	
1	1507 (7.2%)	832 (5.5%)	675 (11.6%)	
Arthritis				<0.001
0	20150 (96.1%)	14588 (96.5%)	5562 (95.2%)	
1	817 (3.9%)	536 (3.5%)	281 (4.8%)	

Table S2. Baseline characteristics of predictors for congnitive impariment

	Total (N=20967)	Non-Cognitive impairment (N=1229)	Cognitive impairment (N=19738)	p.overall
Waist circumference	85.8 (18.13)	84.83 (12.39)	85.86 (18.43)	0.006
Systolic blood pressure (SBP)	132.09 (61.86)	130.93 (62.77)	132.16 (61.8)	0.502
Diastolic blood pressure (DBP)	75.37 (10.37)	74.83 (9.74)	75.4 (10.4)	0.049
Pulse rate	74.12 (9.45)	74.46 (9.46)	74.09 (9.45)	0.185
Peak Expiratory Flow (PEF)	292.33 (118.32)	290.47 (116.22)	292.44 (118.45)	0.563
Left hand grip strength	29.36 (45.36)	27.81 (17.03)	29.46 (46.56)	0.005
Right hand grip strength	30.64 (43.97)	29.9 (32.27)	30.69 (44.6)	0.422
Walking speed	1.68 (3.52)	1.61 (3.34)	1.68 (3.53)	0.458
BMI	23.90(3.92)	23.71(3.94)	23.91(3.92)	0.147
WBC	5.89 (1.63)	5.96 (3.47)	5.89 (1.44)	0.447
Hb	13.66 (1.55)	13.63 (1.5)	13.66 (1.55)	0.518
Hct	41.35 (4.52)	41.33 (4.46)	41.35 (4.52)	0.847
MCV	91.57 (6.19)	91.93 (6.0)	91.55 (6.2)	0.034
PLT	203.62 (60.39)	204.26 (63.46)	203.58 (60.2)	0.714
TG	132.83 (74.01)	128.31 (66.59)	133.11 (74.44)	0.015
Cr	0.79 (0.23)	0.79 (0.18)	0.79 (0.23)	0.354
HDL	50.67 (9.22)	50.59 (8.36)	50.68 (9.27)	0.732
LDL	101.35 (23.14)	101.63 (21.24)	101.33 (23.26)	0.633
TC	182.62 (29.2)	182.24 (27.07)	182.64 (29.33)	0.617
GLU	100.6 (28.4)	99.29 (28.94)	100.68 (28.36)	0.102
UA	4.89 (1.13)	4.93 (1.14)	4.89 (1.13)	0.172
Cys-C	0.84 (0.19)	0.84 (0.16)	0.84 (0.2)	0.51
CRP	2.24 (4.8)	2.1 (3.91)	2.25 (4.85)	0.2
HbA1c	5.91 (0.79)	5.9 (0.79)	5.91 (0.79)	0.541
Age	68.2 (10.47)	68.15 (10.68)	68.2 (10.46)	0.882
Sleep quality	6.4 (1.86)	6.68 (1.39)	6.39 (1.88)	<0.001
Daytime napping	38.0 (43.28)	36.97 (38.77)	38.06 (43.54)	0.342
Activities of daily living (ADL)	5.88 (0.6)	5.99 (0.13)	5.87 (0.62)	<0.001
Upper Arm Length	33.38(2.32)	33.35(2.14)	33.38(2.33)	0.624
Knee Length	47.59(3.08)	47.47(2.92)	47.60(3.09)	0.120
Asthma				0.048
0	20833 (99.4%)	1227 (99.8%)	19606 (99.3%)	
1	134 (0.6%)	2 (0.2%)	132 (0.7%)	
Assistive Device				<0.001

Use				
0	19460 (92.8%)	1212 (98.6%)	18248 (92.5%)	
1	1507 (7.2%)	17 (1.4%)	1490 (7.5%)	
Edentulism / Complete Tooth Loss				<0.001
0	20150 (96.1%)	1205 (98.0%)	18945 (96.0%)	
1	817 (3.9%)	24 (2.0%)	793 (4.0%)	
Standing balance				0.727
1	20877 (99.6%)	1225 (99.7%)	19652 (99.6%)	
0	90 (0.4%)	4 (0.3%)	86 (0.4%)	
Gender				0.239
0	10995 (52.4%)	624 (50.8%)	10371 (52.5%)	
1	9972 (47.6%)	605 (49.2%)	9367 (47.5%)	
Marital status				0.09
1	16919 (80.7%)	959 (78.0%)	15960 (80.9%)	
5	2321 (11.1%)	145 (11.8%)	2176 (11.0%)	
2	1296 (6.2%)	88 (7.2%)	1208 (6.1%)	
3	56 (0.3%)	6 (0.5%)	50 (0.3%)	
4	192 (0.9%)	14 (1.1%)	178 (0.9%)	
6	157 (0.7%)	15 (1.2%)	142 (0.7%)	
7	26 (0.1%)	2 (0.2%)	24 (0.1%)	
Hearing				0.479
0	13561 (64.7%)	777 (63.2%)	12784 (64.8%)	
1	5563 (26.5%)	344 (28.0%)	5219 (26.4%)	
Smoking Status	1843 (8.8%)	108 (8.8%)	1735 (8.8%)	
1				0.75
0	8756 (41.8%)	508 (41.3%)	8248 (41.8%)	
Alcohol consumption	1347 (6.4%)	80 (6.5%)	1267 (6.4%)	
0	3029 (14.4%)	174 (14.2%)	2855 (14.5%)	
2	6923 (33.0%)	414 (33.7%)	6509 (33.0%)	
1	523 (2.5%)	23 (1.9%)	500 (2.5%)	
Social participation	242 (1.2%)	20 (1.6%)	222 (1.1%)	
0	93 (0.4%)	7 (0.6%)	86 (0.4%)	
3	11 (0.1%)	1 (0.1%)	10 (0.1%)	
2	38 (0.2%)	2 (0.2%)	36 (0.2%)	
1	5 (0.0%)	0 (0.0%)	5 (0.0%)	
4				0.223
5	11185 (53.3%)	645 (52.5%)	10540 (53.4%)	
6	4034 (19.2%)	222 (18.1%)	3812 (19.3%)	
8	3755 (17.9%)	226 (18.4%)	3529 (17.9%)	
7	1257 (6.0%)	91 (7.4%)	1166 (5.9%)	
9	736 (3.5%)	45 (3.7%)	691 (3.5%)	
Health satisfaction				0.154
2	11055 (52.7%)	638 (51.9%)	10417 (52.8%)	

3	7231 (34.5%)	447 (36.4%)	6784 (34.4%)	
1	944 (4.5%)	46 (3.7%)	898 (4.5%)	
0	364 (1.7%)	22 (1.8%)	342 (1.7%)	
4	1364 (6.5%)	74 (6.0%)	1290 (6.5%)	
Marital satisfaction	9 (0.0%)	2 (0.2%)	7 (0.0%)	
2				0.576
3	7053 (33.6%)	400 (32.5%)	6653 (33.7%)	
1	10940 (52.2%)	647 (52.6%)	10293 (52.1%)	
0	1325 (6.3%)	82 (6.7%)	1243 (6.3%)	
4	1312 (6.3%)	85 (6.9%)	1227 (6.2%)	
6	337 (1.6%)	15 (1.2%)	322 (1.6%)	
Life satisfaction				0.069
3	20577 (98.1%)	1215 (98.9%)	19362 (98.1%)	
2	390 (1.9%)	14 (1.1%)	376 (1.9%)	
1				0.284
4	15742 (75.1%)	939 (76.4%)	14803 (75.0%)	
0	5225 (24.9%)	290 (23.6%)	4935 (25.0%)	
Prostate medication				0.954
0	19602 (93.5%)	1148 (93.4%)	18454 (93.5%)	
1	1365 (6.5%)	81 (6.6%)	1284 (6.5%)	
Antihypertensive medication				0.091
0	20804 (99.2%)	1225 (99.7%)	19579 (99.2%)	
1	163 (0.8%)	4 (0.3%)	159 (0.8%)	
Diabetes medication				<0.001
0	14956 (71.3%)	824 (67.0%)	14132 (71.6%)	
1	6011 (28.7%)	405 (33.0%)	5606 (28.4%)	
Cancer medication				<0.001
0	15124 (72.1%)	1033 (84.1%)	14091 (71.4%)	
1	5843 (27.9%)	196 (15.9%)	5647 (28.6%)	
Analgesic use				0.084
0	20756 (99.0%)	1223 (99.5%)	19533 (99.0%)	
1	211 (1.0%)	6 (0.5%)	205 (1.0%)	
Pain severity				0.011
0	20710 (98.8%)	1224 (99.6%)	19486 (98.7%)	
1	257 (1.2%)	5 (0.4%)	252 (1.3%)	
Cataract surgery				<0.001
0	17208 (82.1%)	1081 (88.0%)	16127 (81.7%)	
1	3759 (17.9%)	148 (12.0%)	3611 (18.3%)	
Glaucoma				<0.001
0	17156 (81.8%)	1120 (91.1%)	16036 (81.2%)	
1	3811 (18.2%)	109 (8.9%)	3702 (18.8%)	
Depression status				0.68
0	18732 (89.3%)	1091 (88.8%)	17641 (89.4%)	

1	2169 (10.3%)	135 (11.0%)	2034 (10.3%)	
2	66 (0.3%)	3 (0.2%)	63 (0.3%)	
Myopia				<0.001
0	18050 (86.1%)	1153 (93.8%)	16897 (85.6%)	
1	2917 (13.9%)	76 (6.2%)	2841 (14.4%)	
Hyperopia				0.958
0	19569 (93.3%)	1148 (93.4%)	18421 (93.3%)	
1	1398 (6.7%)	81 (6.6%)	1317 (6.7%)	
Cognitive impairment				0.048
1	20065 (95.7%)	1162 (94.5%)	18903 (95.8%)	
0	902 (4.3%)	67 (5.5%)	835 (4.2%)	
Disability status				0.131
0	20401 (97.3%)	1187 (96.6%)	19214 (97.3%)	
1	566 (2.7%)	42 (3.4%)	524 (2.7%)	
Hypertension				0.976
0	20872 (99.5%)	1224 (99.6%)	19648 (99.5%)	
1	95 (0.5%)	5 (0.4%)	90 (0.5%)	
Dyslipidemia				0.105
0	20535 (97.9%)	1212 (98.6%)	19323 (97.9%)	
1	432 (2.1%)	17 (1.4%)	415 (2.1%)	
Diabetes mellitus				0.698
0	20712 (98.8%)	1216 (98.9%)	19496 (98.8%)	
1	255 (1.2%)	13 (1.1%)	242 (1.2%)	
Malignant tumor				0.217
0	20248 (96.6%)	1195 (97.2%)	19053 (96.5%)	
1	719 (3.4%)	34 (2.8%)	685 (3.5%)	
Lung disease				0.207
0	20810 (99.3%)	1224 (99.6%)	19586 (99.2%)	
1	157 (0.7%)	5 (0.4%)	152 (0.8%)	
Liver disease				0.267
0	20582 (98.2%)	1212 (98.6%)	19370 (98.1%)	
1	385 (1.8%)	17 (1.4%)	368 (1.9%)	
Heart disease				0.729
0	20311 (96.9%)	1188 (96.7%)	19123 (96.9%)	
1	656 (3.1%)	41 (3.3%)	615 (3.1%)	
Stroke				0.115
0	20884 (99.6%)	1228 (99.9%)	19656 (99.6%)	
1	83 (0.4%)	1 (0.1%)	82 (0.4%)	
Kidney disease				0.047
0	20807 (99.2%)	1226 (99.8%)	19581 (99.2%)	
1	160 (0.8%)	3 (0.2%)	157 (0.8%)	
Stomach disease				0.168
0	20307 (96.9%)	1199 (97.6%)	19108 (96.8%)	
1	660 (3.1%)	30 (2.4%)	630 (3.2%)	
Affective disorder				0.048
0	20833 (99.4%)	1227 (99.8%)	19606 (99.3%)	

1	134 (0.6%)	2 (0.2%)	132 (0.7%)	
Memory-related disease				<0.001
0	19460 (92.8%)	1212 (98.6%)	18248 (92.5%)	
1	1507 (7.2%)	17 (1.4%)	1490 (7.5%)	
Arthritis				<0.001
0	20150 (96.1%)	1205 (98.0%)	18945 (96.0%)	
1	817 (3.9%)	24 (2.0%)	793 (4.0%)	

Table S3. Baseline characteristics of predictors for hearing

	**Total (N=20967)**	**Non-**Hearing Impairment **(N=13708)**	Hearing Impairment **(N=7259)**	**p.overall**
Waist circumference	85.8 (18.13)	85.78 (17.67)	85.84 (18.98)	0.834
Systolic blood pressure (SBP)	132.09 (61.86)	131.89 (60.08)	132.47 (65.07)	0.525
Diastolic blood pressure (DBP)	75.37 (10.37)	75.33 (10.27)	75.44 (10.55)	0.478
Pulse rate	74.12 (9.45)	74.1 (9.41)	74.15 (9.52)	0.685
Peak Expiratory Flow (PEF)	292.33(118.32)	292.6 (118.73)	291.81(117.54)	0.646
Left hand grip strength	29.36 (45.36)	29.44 (46.67)	29.2 (42.8)	0.703
Right hand grip strength	30.64 (43.97)	30.77 (45.38)	30.39 (41.18)	0.539
Walking speed	1.68 (3.52)	1.67 (3.51)	1.69 (3.55)	0.718
BMI	23.90(3.92)	23.90(3.96)	23.90(3.85)	0.988
WBC	5.89 (1.63)	5.89 (1.45)	5.91 (1.93)	0.386
Hb	13.66 (1.55)	13.66 (1.54)	13.65 (1.55)	0.583
Hct	41.35 (4.52)	41.36 (4.5)	41.34 (4.54)	0.71
MCV	91.57 (6.19)	91.63 (6.18)	91.47 (6.2)	0.067
PLT	203.62 (60.39)	204.03 (61.33)	202.85 (58.57)	0.174
TG	132.83 (74.01)	132.29 (73.38)	133.84 (75.19)	0.152
Cr	0.79 (0.23)	0.79 (0.21)	0.79 (0.26)	0.096
HDL	50.67 (9.22)	50.73 (9.31)	50.57 (9.04)	0.233
LDL	101.35 (23.14)	101.06 (22.94)	101.89 (23.52)	0.014
TC	182.62 (29.2)	182.27 (28.91)	183.27 (29.73)	0.019
GLU	100.6 (28.4)	100.49 (27.75)	100.8 (29.58)	0.466
UA	4.89 (1.13)	4.89 (1.12)	4.89 (1.15)	0.991
Cys-C	0.84 (0.19)	0.84 (0.19)	0.84 (0.2)	0.773
CRP	2.24 (4.8)	2.2 (4.58)	2.32 (5.19)	0.101
HbA1c	5.91 (0.79)	5.91 (0.78)	5.92 (0.82)	0.23
Age	68.2 (10.47)	68.24 (10.43)	68.11 (10.55)	0.403
Sleep quality	6.4 (1.86)	6.28 (1.87)	6.63 (1.8)	<0.001
Daytime napping	38.0 (43.28)	37.74 (42.89)	38.48 (44.0)	0.242
Activities of daily living (ADL)	5.88 (0.6)	5.84 (0.68)	5.94 (0.42)	<0.001

Upper Arm Length	33.38(2.32)	33.40(2.32)	33.35(2.32)	0.156
Knee Length	47.59(3.08)	47.62(3.05)	47.55(3.13)	0.134
Asthma				0.002
0	20833 (99.4%)	13603 (99.2%)	7230 (99.6%)	
1	134 (0.6%)	105 (0.8%)	29 (0.4%)	
Assistive Device Use				<0.001
0	19460 (92.8%)	12517 (91.3%)	6943 (95.6%)	
1	1507 (7.2%)	1191 (8.7%)	316 (4.4%)	
Edentulism / Complete Tooth Loss				<0.001
0	20150 (96.1%)	13112 (95.7%)	7038 (97.0%)	
1	817 (3.9%)	596 (4.3%)	221 (3.0%)	
Standing balance				0.713
1	20877 (99.6%)	13647 (99.6%)	7230 (99.6%)	
0	90 (0.4%)	61 (0.4%)	29 (0.4%)	
Gender				0.524
0	10995 (52.4%)	7166 (52.3%)	3829 (52.7%)	
1	9972 (47.6%)	6542 (47.7%)	3430 (47.3%)	
Marital status				0.899
1	16919 (80.7%)	11051 (80.6%)	5868 (80.8%)	
5	2321 (11.1%)	1513 (11.0%)	808 (11.1%)	
2	1296 (6.2%)	866 (6.3%)	430 (5.9%)	
3	56 (0.3%)	35 (0.3%)	21 (0.3%)	
4	192 (0.9%)	128 (0.9%)	64 (0.9%)	
6	157 (0.7%)	98 (0.7%)	59 (0.8%)	
7	26 (0.1%)	17 (0.1%)	9 (0.1%)	
Hearing				0.99
0	8486 (40.5%)	5549 (40.5%)	2937 (40.5%)	
1	12481 (59.5%)	8159 (59.5%)	4322 (59.5%)	
Smoking Status				0.853
1	13561 (64.7%)	8872 (64.7%)	4689 (64.6%)	
0	5563 (26.5%)	3642 (26.6%)	1921 (26.5%)	
Alcohol consumption	1843 (8.8%)	1194 (8.7%)	649 (8.9%)	
0				0.447
2	8756 (41.8%)	5798 (42.3%)	2958 (40.7%)	
1	1347 (6.4%)	861 (6.3%)	486 (6.7%)	
Social participation	3029 (14.4%)	1992 (14.5%)	1037 (14.3%)	
0	6923 (33.0%)	4476 (32.7%)	2447 (33.7%)	
3	523 (2.5%)	326 (2.4%)	197 (2.7%)	
2	242 (1.2%)	159 (1.2%)	83 (1.1%)	
1	93 (0.4%)	63 (0.5%)	30 (0.4%)	
4	11 (0.1%)	6 (0.0%)	5 (0.1%)	
5	38 (0.2%)	24 (0.2%)	14 (0.2%)	
6	5 (0.0%)	3 (0.0%)	2 (0.0%)	

8				0.934
7	11185 (53.3%)	7317 (53.4%)	3868 (53.3%)	
9	4034 (19.2%)	2641 (19.3%)	1393 (19.2%)	
Health satisfaction	3755 (17.9%)	2456 (17.9%)	1299 (17.9%)	
2	1257 (6.0%)	808 (5.9%)	449 (6.2%)	
3	736 (3.5%)	486 (3.5%)	250 (3.4%)	
1				0.936
0	11055 (52.7%)	7220 (52.7%)	3835 (52.8%)	
4	7231 (34.5%)	4746 (34.6%)	2485 (34.2%)	
Marital satisfaction	944 (4.5%)	613 (4.5%)	331 (4.6%)	
2	364 (1.7%)	231 (1.7%)	133 (1.8%)	
3	1364 (6.5%)	893 (6.5%)	471 (6.5%)	
1	9 (0.0%)	5 (0.0%)	4 (0.1%)	
0				0.036
4	7053 (33.6%)	4574 (33.4%)	2479 (34.2%)	
6	10940 (52.2%)	7115 (51.9%)	3825 (52.7%)	
Life satisfaction	1325 (6.3%)	909 (6.6%)	416 (5.7%)	
3	1312 (6.3%)	883 (6.4%)	429 (5.9%)	
2	337 (1.6%)	227 (1.7%)	110 (1.5%)	
1				<0.001
4	20577 (98.1%)	13412 (97.8%)	7165 (98.7%)	
0	390 (1.9%)	296 (2.2%)	94 (1.3%)	
Prostate medication				0.451
0	15742 (75.1%)	10269 (74.9%)	5473 (75.4%)	
1	5225 (24.9%)	3439 (25.1%)	1786 (24.6%)	
Antihypertensive medication				0.996
0	19602 (93.5%)	12815 (93.5%)	6787 (93.5%)	
1	1365 (6.5%)	893 (6.5%)	472 (6.5%)	
Diabetes medication				0.327
0	20804 (99.2%)	13595 (99.2%)	7209 (99.3%)	
1	163 (0.8%)	113 (0.8%)	50 (0.7%)	
Cancer medication				0.738
0	14956 (71.3%)	9789 (71.4%)	5167 (71.2%)	
1	6011 (28.7%)	3919 (28.6%)	2092 (28.8%)	
Analgesic use				<0.001
0	15124 (72.1%)	9287 (67.7%)	5837 (80.4%)	
1	5843 (27.9%)	4421 (32.3%)	1422 (19.6%)	
Pain severity				0.004
0	20756 (99.0%)	13550 (98.8%)	7206 (99.3%)	
1	211 (1.0%)	158 (1.2%)	53 (0.7%)	
Cataract surgery				<0.001
0	20710 (98.8%)	13514 (98.6%)	7196 (99.1%)	
1	257 (1.2%)	194 (1.4%)	63 (0.9%)	
Glaucoma				0.557
0	18732 (89.3%)	12226 (89.2%)	6506 (89.6%)	

1	2169 (10.3%)	1440 (10.5%)	729 (10.0%)	
Depression status	66 (0.3%)	42 (0.3%)	24 (0.3%)	
0				<0.001
1	17208 (82.1%)	10844 (79.1%)	6364 (87.7%)	
2	3759 (17.9%)	2864 (20.9%)	895 (12.3%)	
Myopia				<0.001
0	17156 (81.8%)	10671 (77.8%)	6485 (89.3%)	
1	3811 (18.2%)	3037 (22.2%)	774 (10.7%)	
Hyperopia				<0.001
0	19738 (94.1%)	13166 (96.0%)	6572 (90.5%)	
1	1229 (5.9%)	542 (4.0%)	687 (9.5%)	
Cognitive impairment				<0.001
1	18050 (86.1%)	11271 (82.2%)	6779 (93.4%)	
0	2917 (13.9%)	2437 (17.8%)	480 (6.6%)	
Disability status				0.029
0	19569 (93.3%)	12756 (93.1%)	6813 (93.9%)	
1	1398 (6.7%)	952 (6.9%)	446 (6.1%)	
Hypertension				0.656
0	20065 (95.7%)	13125 (95.7%)	6940 (95.6%)	
1	902 (4.3%)	583 (4.3%)	319 (4.4%)	
Dyslipidemia				0.005
0	20401 (97.3%)	13306 (97.1%)	7095 (97.7%)	
1	566 (2.7%)	402 (2.9%)	164 (2.3%)	
Diabetes mellitus				0.244
0	20872 (99.5%)	13640 (99.5%)	7232 (99.6%)	
1	95 (0.5%)	68 (0.5%)	27 (0.4%)	
Malignant tumor				<0.001
0	20535 (97.9%)	13375 (97.6%)	7160 (98.6%)	
1	432 (2.1%)	333 (2.4%)	99 (1.4%)	
Lung disease				0.245
0	20712 (98.8%)	13532 (98.7%)	7180 (98.9%)	
1	255 (1.2%)	176 (1.3%)	79 (1.1%)	
Liver disease				<0.001
0	20248 (96.6%)	13188 (96.2%)	7060 (97.3%)	
1	719 (3.4%)	520 (3.8%)	199 (2.7%)	
Heart disease				0.005
0	20810 (99.3%)	13588 (99.1%)	7222 (99.5%)	
1	157 (0.7%)	120 (0.9%)	37 (0.5%)	
Stroke				<0.001
0	20582 (98.2%)	13425 (97.9%)	7157 (98.6%)	
1	385 (1.8%)	283 (2.1%)	102 (1.4%)	
Kidney disease				0.026
0	20311 (96.9%)	13252 (96.7%)	7059 (97.2%)	
1	656 (3.1%)	456 (3.3%)	200 (2.8%)	
Stomach disease				0.009
0	20884 (99.6%)	13642 (99.5%)	7242 (99.8%)	
1	83 (0.4%)	66 (0.5%)	17 (0.2%)	
Affective disorder				<0.001

0	20807 (99.2%)	13574 (99.0%)	7233 (99.6%)	
1	160 (0.8%)	134 (1.0%)	26 (0.4%)	
Memory-related disease				0.319
0	20307 (96.9%)	13264 (96.8%)	7043 (97.0%)	
1	660 (3.1%)	444 (3.2%)	216 (3.0%)	
Arthritis				0.002
0	20833 (99.4%)	13603 (99.2%)	7230 (99.6%)	
1	134 (0.6%)	105 (0.8%)	29 (0.4%)	
Waist circumference				<0.001
Systolic blood pressure (SBP)	19460 (92.8%)	12517 (91.3%)	6943 (95.6%)	
Diastolic blood pressure (DBP)	1507 (7.2%)	1191 (8.7%)	316 (4.4%)	
Pulse rate				<0.001
Respiratory rate	20150 (96.1%)	13112 (95.7%)	7038 (97.0%)	
Left hand grip strength	817 (3.9%)	596 (4.3%)	221 (3.0%)	

Table S4. Baseline characteristics of predictors for depression

	Total (N=20967)	Non-Depression (N=18732)	Depression (N=2169)	p.overall
Waist circumference	85.8 (18.13)	85.95 (18.72)	84.64 (12.0)	<0.001
Systolic blood pressure (SBP)	132.09 (61.86)	132.15 (61.13)	130.89 (63.34)	0.38
Diastolic blood pressure (DBP)	75.37 (10.37)	75.47 (10.32)	74.53 (10.67)	<0.001
Pulse rate	74.12 (9.45)	74.08 (9.39)	74.38 (9.93)	0.184
Peak Expiratory Flow (PEF)	292.33 (118.32)	295.41 (117.95)	267.22 (118.98)	<0.001
Left hand grip strength	29.36 (45.36)	29.45 (42.33)	28.74 (66.42)	0.628
Right hand grip strength	30.64 (43.97)	30.96 (43.05)	28.11 (51.7)	0.014
Walking speed	1.68 (3.52)	1.66 (3.65)	1.82 (2.12)	0.002
BMI	23.90(3.92)	23.86(3.89)	24.00(4.00)	0.049
WBC	5.89 (1.63)	5.89 (1.43)	5.92 (2.83)	0.642
Hb	13.66 (1.55)	13.69 (1.54)	13.41 (1.61)	<0.001
Hct	41.35 (4.52)	41.44 (4.5)	40.61 (4.61)	<0.001
MCV	91.57 (6.19)	91.62 (6.15)	91.14 (6.52)	0.001
PLT	203.62 (60.39)	203.38 (60.13)	205.6 (62.92)	0.119
TG	132.83 (74.01)	132.8 (73.7)	132.78 (75.8)	0.995
Cr	0.79 (0.23)	0.79 (0.23)	0.77 (0.26)	<0.001
HDL	50.67 (9.22)	50.6 (9.15)	51.18 (9.68)	0.008
LDL	101.35 (23.14)	101.31 (22.9)	101.64 (25.07)	0.558
TC	182.62 (29.2)	182.52 (28.89)	183.29 (31.63)	0.28
GLU	100.6 (28.4)	100.45 (27.53)	101.9 (35.16)	0.063
UA	4.89 (1.13)	4.91 (1.13)	4.68 (1.13)	<0.001
Cys-C	0.84 (0.19)	0.84 (0.19)	0.84 (0.22)	0.952

CRP	2.24 (4.8)	2.22 (4.69)	2.49 (5.74)	0.029
HbA1c	5.91 (0.79)	5.91 (0.77)	5.96 (0.97)	0.013
Age	68.2 (10.47)	68.16 (10.54)	68.49 (9.92)	0.146
Sleep quality	6.4 (1.86)	6.4 (1.86)	6.4 (1.8)	0.956
Daytime napping	38.0 (43.28)	37.66 (43.21)	40.41 (43.44)	0.005
Activities of daily living (ADL)	5.88 (0.6)	5.88 (0.6)	5.88 (0.6)	0.624
Upper Arm Length	33.38(2.32)	33.44(2.31)	32.86(2.28)	<0.001
Knee Length	47.59(3.08)	47.67(3.06)	46.91(3.14)	<0.001
Asthma				1.000
0	20833 (99.4%)	18678 (99.4%)	2155 (99.4%)	
1	134 (0.6%)	120 (0.6%)	14 (0.6%)	
Assistive Device Use				0.461
0	19460 (92.8%)	17438 (92.8%)	2022 (93.2%)	
1	1507 (7.2%)	1360 (7.2%)	147 (6.8%)	
Edentulism / Complete Tooth Loss				0.557
0	20150 (96.1%)	18060 (96.1%)	2090 (96.4%)	
1	817 (3.9%)	738 (3.9%)	79 (3.6%)	
Standing balance				0.4
1	20877 (99.6%)	18652 (99.6%)	2160 (99.6%)	
0	90 (0.4%)	80 (0.4%)	9 (0.4%)	
Gender				<0.001
0	10995 (52.4%)	9451 (50.5%)	1500 (69.2%)	
1	9972 (47.6%)	9281 (49.5%)	669 (30.8%)	
Marital status				<0.001
1	16919 (80.7%)	15259 (81.5%)	1618 (74.6%)	
5	2321 (11.1%)	1965 (10.5%)	343 (15.8%)	
2	1296 (6.2%)	1135 (6.1%)	154 (7.1%)	
3	56 (0.3%)	44 (0.2%)	12 (0.6%)	
4	192 (0.9%)	169 (0.9%)	22 (1.0%)	
6	157 (0.7%)	137 (0.7%)	17 (0.8%)	
7	26 (0.1%)	23 (0.1%)	3 (0.1%)	
Hearing				<0.001
0	13561 (64.7%)	11929 (63.7%)	1584 (73.0%)	
1	5563 (26.5%)	5140 (27.4%)	407 (18.8%)	
Smoking Status	1843 (8.8%)	1663 (8.9%)	178 (8.2%)	
1				<0.001
0	8756 (41.8%)	7634 (40.8%)	1078 (49.7%)	
Alcohol consumption	1347 (6.4%)	1232 (6.6%)	114 (5.3%)	
0	3029 (14.4%)	2710 (14.5%)	315 (14.5%)	
2	6923 (33.0%)	6293 (33.6%)	614 (28.3%)	
1	523 (2.5%)	492 (2.6%)	30 (1.4%)	
Social participation	242 (1.2%)	229 (1.2%)	13 (0.6%)	

0	93 (0.4%)	91 (0.5%)	2 (0.1%)	
3	11 (0.1%)	9 (0.0%)	2 (0.1%)	
2	38 (0.2%)	37 (0.2%)	1 (0.0%)	
1	5 (0.0%)	5 (0.0%)	0 (0.0%)	
4				<0.001
5	11185 (53.3%)	10474 (55.9%)	696 (32.1%)	
6	4034 (19.2%)	3727 (19.9%)	299 (13.8%)	
8	3755 (17.9%)	3051 (16.3%)	681 (31.4%)	
7	1257 (6.0%)	787 (4.2%)	450 (20.7%)	
9	736 (3.5%)	693 (3.7%)	43 (2.0%)	
Health satisfaction				<0.001
2	11055 (52.7%)	9871 (52.7%)	1160 (53.5%)	
3	7231 (34.5%)	6595 (35.2%)	618 (28.5%)	
1	944 (4.5%)	727 (3.9%)	201 (9.3%)	
0	364 (1.7%)	253 (1.4%)	105 (4.8%)	
4	1364 (6.5%)	1279 (6.8%)	83 (3.8%)	
Marital satisfaction	9 (0.0%)	7 (0.0%)	2 (0.1%)	
2				<0.001
3	7053 (33.6%)	6483 (34.6%)	549 (25.3%)	
1	10940 (52.2%)	9890 (52.8%)	1028 (47.4%)	
0	1325 (6.3%)	961 (5.1%)	348 (16.0%)	
4	1312 (6.3%)	1224 (6.5%)	87 (4.0%)	
6	337 (1.6%)	174 (0.9%)	157 (7.2%)	
Life satisfaction				0.763
3	20577 (98.1%)	18383 (98.1%)	2130 (98.2%)	
2	390 (1.9%)	349 (1.9%)	39 (1.8%)	
1				<0.001
4	15742 (75.1%)	14169 (75.6%)	1531 (70.6%)	
0	5225 (24.9%)	4563 (24.4%)	638 (29.4%)	
Prostate medication				<0.001
0	19602 (93.5%)	17582 (93.9%)	1957 (90.2%)	
1	1365 (6.5%)	1150 (6.1%)	212 (9.8%)	
Antihypertensive medication				0.742
0	20804 (99.2%)	18587 (99.2%)	2151 (99.2%)	
1	163 (0.8%)	145 (0.8%)	18 (0.8%)	
Diabetes medication				<0.001
0	14956 (71.3%)	13731 (73.3%)	1191 (54.9%)	
1	6011 (28.7%)	5001 (26.7%)	978 (45.1%)	
Cancer medication				0.876
0	15124 (72.1%)	13522 (72.2%)	1555 (71.7%)	
1	5843 (27.9%)	5210 (27.8%)	614 (28.3%)	
Analgesic use				0.015
0	20756 (99.0%)	18547 (99.0%)	2146 (98.9%)	
1	211 (1.0%)	185 (1.0%)	23 (1.1%)	
Pain severity				0.628

0	20710 (98.8%)	18498 (98.8%)	2147 (99.0%)	
1	257 (1.2%)	234 (1.2%)	22 (1.0%)	
Cataract surgery				0.211
0	17208 (82.1%)	15358 (82.0%)	1800 (83.0%)	
1	3759 (17.9%)	3374 (18.0%)	369 (17.0%)	
Glaucoma				0.219
0	17156 (81.8%)	15299 (81.7%)	1804 (83.2%)	
1	3811 (18.2%)	3433 (18.3%)	365 (16.8%)	
Depression status				0.68
0	19738 (94.1%)	17641 (94.2%)	2034 (93.8%)	
1	1229 (5.9%)	1091 (5.8%)	135 (6.2%)	
2				0.578
Myopia	18050 (86.1%)	16115 (86.0%)	1880 (86.7%)	
0	2917 (13.9%)	2617 (14.0%)	289 (13.3%)	
1				0.785
Hyperopia	19569 (93.3%)	17490 (93.4%)	2017 (93.0%)	
0	1398 (6.7%)	1242 (6.6%)	152 (7.0%)	
1				0.533
Cognitive impairment	20065 (95.7%)	17935 (95.7%)	2068 (95.3%)	
1	902 (4.3%)	797 (4.3%)	101 (4.7%)	
0				0.216
Disability status	20401 (97.3%)	18225 (97.3%)	2114 (97.5%)	
0	566 (2.7%)	507 (2.7%)	55 (2.5%)	
1				0.4
Hypertension	20872 (99.5%)	18649 (99.6%)	2158 (99.5%)	
0	95 (0.5%)	83 (0.4%)	11 (0.5%)	
1				0.797
Dyslipidemia	20535 (97.9%)	18349 (98.0%)	2122 (97.8%)	
0	432 (2.1%)	383 (2.0%)	47 (2.2%)	
1				0.921
Diabetes mellitus	20712 (98.8%)	18506 (98.8%)	2141 (98.7%)	
0	255 (1.2%)	226 (1.2%)	28 (1.3%)	
1				0.471
Malignant tumor	20248 (96.6%)	18094 (96.6%)	2092 (96.4%)	
0	719 (3.4%)	638 (3.4%)	77 (3.6%)	
1				<0.001
Lung disease	20810 (99.3%)	18596 (99.3%)	2152 (99.2%)	
0	157 (0.7%)	136 (0.7%)	17 (0.8%)	
1				0.068
Liver disease	20582 (98.2%)	18399 (98.2%)	2117 (97.6%)	
0	385 (1.8%)	333 (1.8%)	52 (2.4%)	
1				0.22
Heart disease	20311 (96.9%)	18157 (96.9%)	2089 (96.3%)	
0	656 (3.1%)	575 (3.1%)	80 (3.7%)	
1				0.301
Stroke	20884 (99.6%)	18657 (99.6%)	2162 (99.7%)	
0	83 (0.4%)	75 (0.4%)	7 (0.3%)	
1				0.377
Kidney disease	20807 (99.2%)	18584 (99.2%)	2157 (99.4%)	

0	160 (0.8%)	148 (0.8%)	12 (0.6%)	
1				0.671
Stomach disease	20307 (96.9%)	18145 (96.9%)	2097 (96.7%)	
0	660 (3.1%)	587 (3.1%)	72 (3.3%)	
1				<0.001
Affective disorder	20833 (99.4%)	18616 (99.4%)	2155 (99.4%)	
0	134 (0.6%)	116 (0.6%)	14 (0.6%)	
1				0.225
Memory-related disease	19460 (92.8%)	17380 (92.8%)	2022 (93.2%)	
0	1507 (7.2%)	1352 (7.2%)	147 (6.8%)	
1				0.485
Arthritis	20150 (96.1%)	17995 (96.1%)	2090 (96.4%)	
0	817 (3.9%)	737 (3.9%)	79 (3.6%)	
